# Supplementary material for: Mechanistic basis of teichoic acid transport by a gatekeeper flippase
Source: Nat Commun. 2026 May 25;17:6809. doi: 10.1038/s41467-026-73616-w (PMC13385700; doi:10.1038/s41467-026-73616-w)
Supplement: Supplementary file 1 — Supplementary Information [file 41467_2026_73616_MOESM1_ESM.pdf]

Supplementary materials for

Mechanistic basis of teichoic acid transport by a gatekeeper flippase

Gonzalo Cebrero[†], Amrutha H. Chidananda[†], Eric Cester[†], Julien Dénéréaz[†], Elif Sena Demir, Alen T. Mathew, D. Ryan Bhowmik, Mario de Capitani, Jean-Louis Reymond, Natarajan Kannan, Fikri Y Avci, Jan-Willem Veening[*], Ahmad Reza Mehdipour[*], Camilo Perez[*]

[†]Contributed equally to this work

*Correspondence to: jan-willem.veening@unil.ch, AhmadReza.Mehdipour@UGent.be, camilo.perez@uga.edu

This PDF file includes:

Supplementary Figures 1 to 10

Supplementary Tables 1 to 3

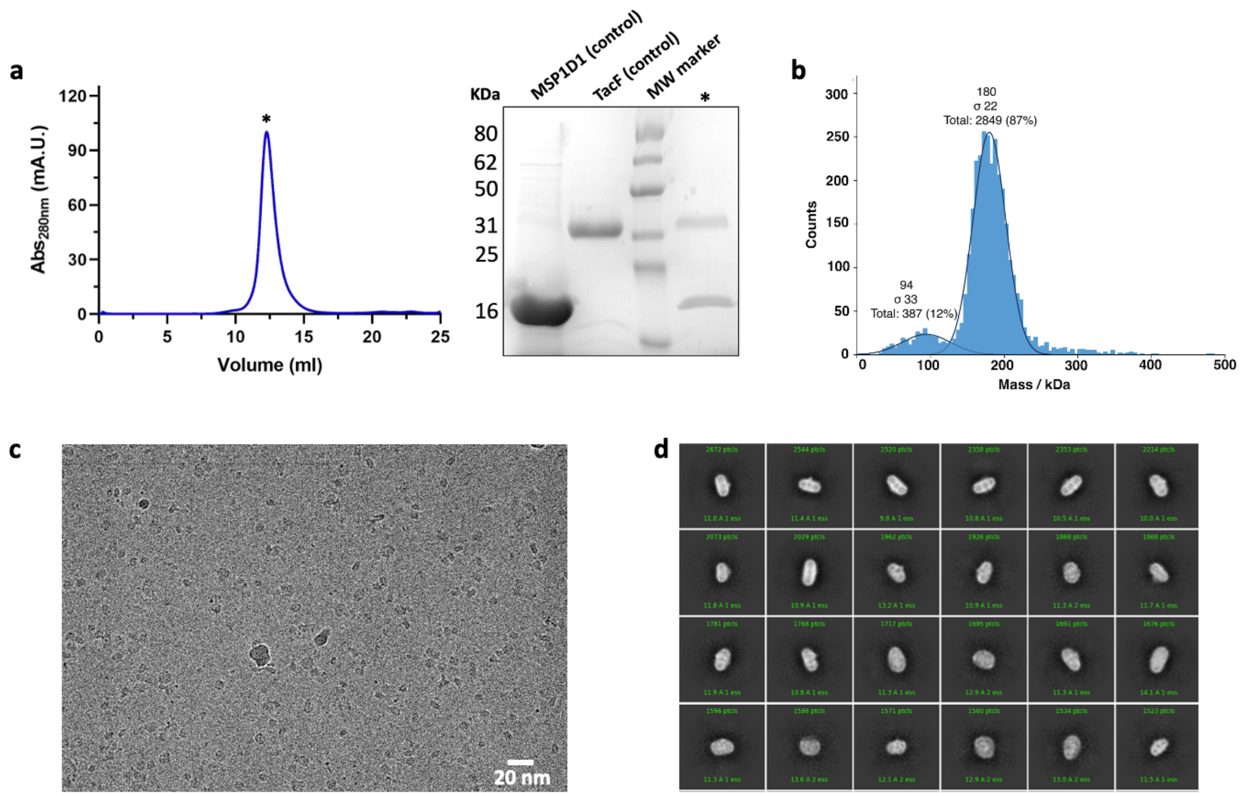

Supplementary Figure 1. **Purification and cryo-EM analysis of TacF wild-type**. **A**. Size exclusion chromatography profile in a Superdex 200 Increase 10/300 column for TacF reconstituted in MSP1D1 nanodiscs. SDS-PAGE of the main peak and controls (purified MSP1D1 and TacF) is shown. **B**. Mass photometry analysis of TacF reconstituted in MSP1D1 nanodiscs. The two distinct populations indicate empty nanodiscs (94 +/- 33kDa) and TacF reconstituted nanodiscs 180 +/- 22kDa). **C**. Representative micrograph of TacF nanodiscs. **D**. 2D class averages of TacF nanodiscs.

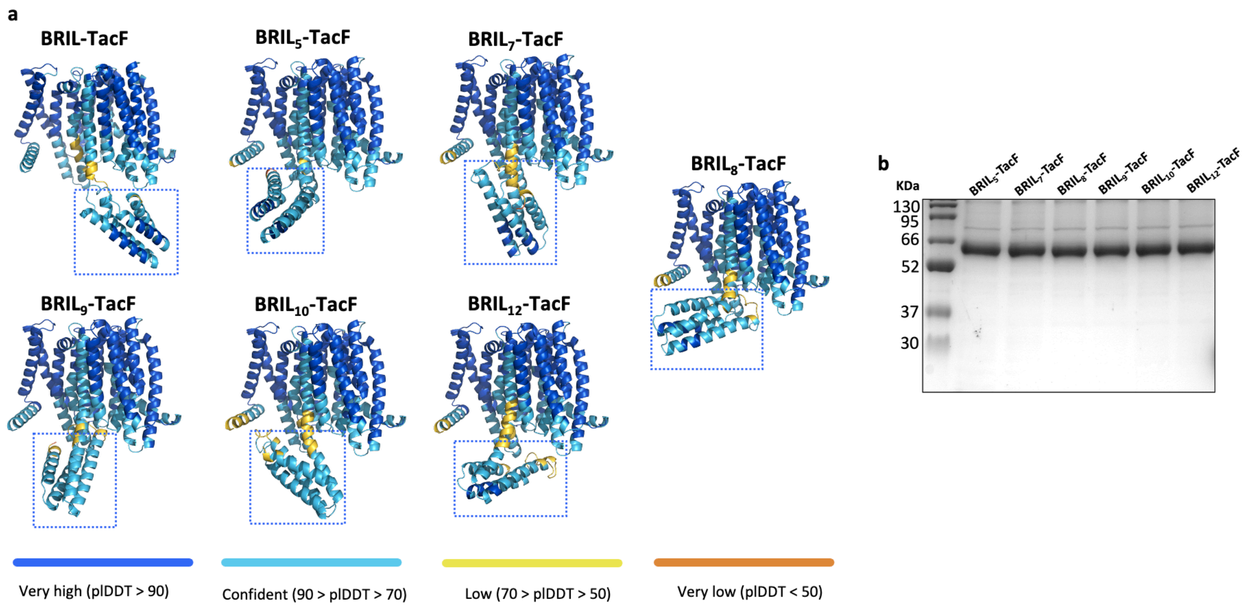

Supplementary Figure 2. ***In silico*** **analysis of BRIL-TacF constructs**. **A**. AlphaFold-3 predicted models of BRIL–TacF fusion constructs. The predicted local distance difference test (pLDDT) indicates local confidence per residue. The blue dotted squares indicate the BRIL portion of the model. **B**. SDS-PAGE analysis of purified BRIL-TacF constructs.

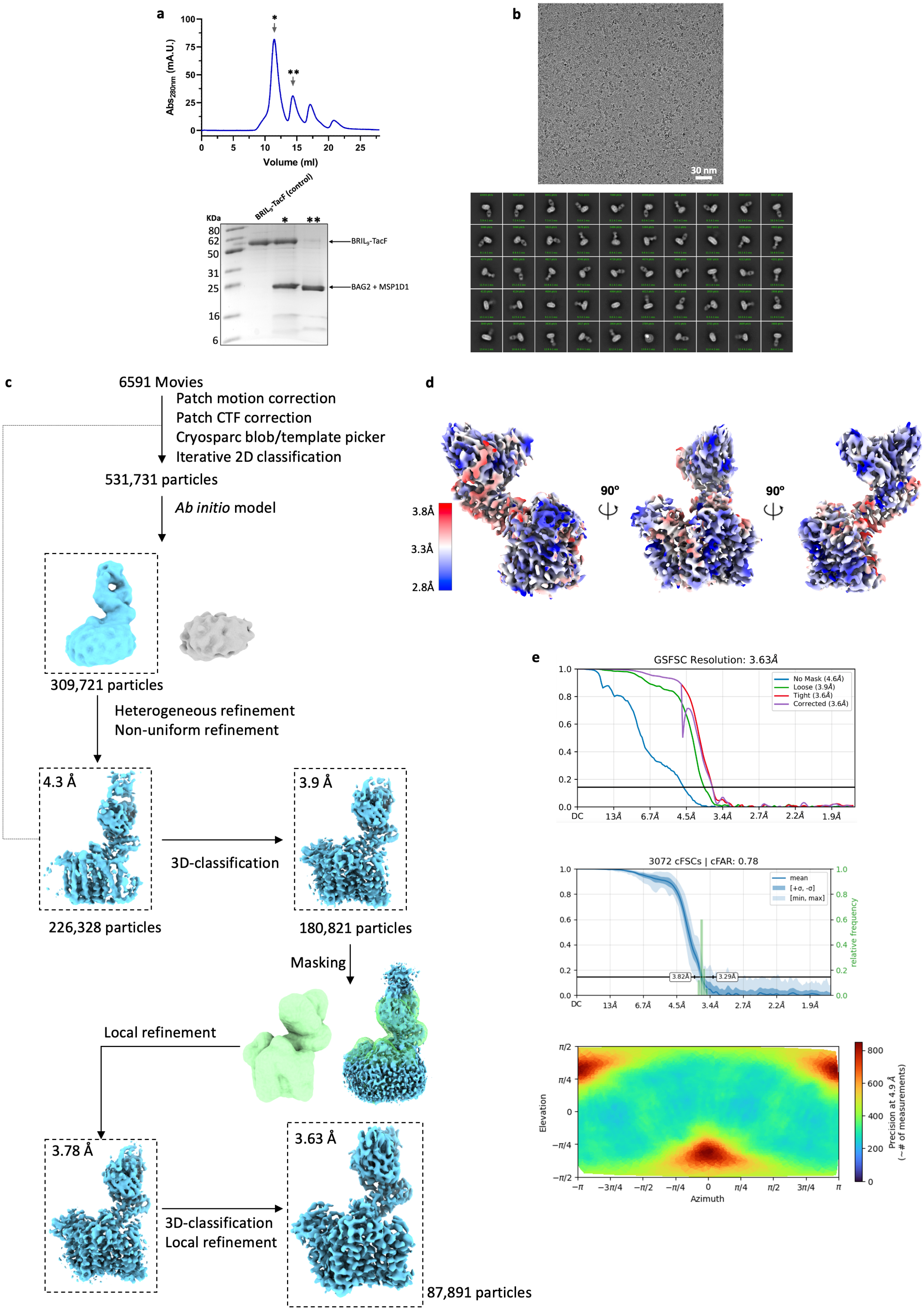
a
Abs280nm (mA.U.)
Volume (ml)
*
**
KDa
BRIL3-TacF (control)
BRIL3-TacF
BAG2 + MSP1D1
b
30 nm
c
6591 Movies
Patch motion correction
Patch CTF correction
Cryosparc blob/template picker
Iterative 2D classification
531,731 particles
Ab initio model
309,721 particles
Heterogeneous refinement
Non-uniform refinement
4.3 Å
226,328 particles
3D-classification
3.9 Å
180,821 particles
Masking
Local refinement
3.78 Å
3D-classification
Local refinement
3.63 Å
87,891 particles
d
3.8Å
3.3Å
2.8Å
90º
90º
e
GSFSC Resolution: 3.63Å
No Mask (4.6Å)
Loose (3.9Å)
Tight (3.6Å)
Corrected (3.6Å)
3072 cFSCs | cFAR: 0.78
mean
[+σ, -σ]
[min, max]
relative frequency
3.82Å
3.29Å
Elevation
Azimuth
Precision at 4.9 Å
(~# of measurements)

Supplementary Figure 3. Cryo-EM characterization of BRIL$_9$-TacF in lipid nanodiscs. **A**. Size exclusion chromatography profile in a Superdex 200 Increase 10/300 column for BRIL$_9$-TacF:BAG2 complex reconstituted in MSP1D1 nanodiscs. SDS-PAGE of the main peaks are shown. **B**. Representative micrograph of BRIL9-TacF:BAG2 nanodiscs and 2D class averages. **C**. Cryo-EM data processing workflow. A set of 6,591 movies was processed, resulting in a set of 531,731 particles that was used as input to generate an *ab initio* reconstruction volume, which was refined through heterogeneous and non-uniform refinement. The resulting volume was used to pick a new set of particles, which were further processed using 3D classification, local refinements, and masking of the nanodisc and part of the BAG2 Fab. This resulted in a 3.63 Å local resolution map. **D**. Local resolution map of the final volume computed in cryoSPARC. **E**. GSFSC and cFSC curves used for resolution estimation of the final map. *Bottom*, Angular viewing directions distribution of particles contributing to the final map.

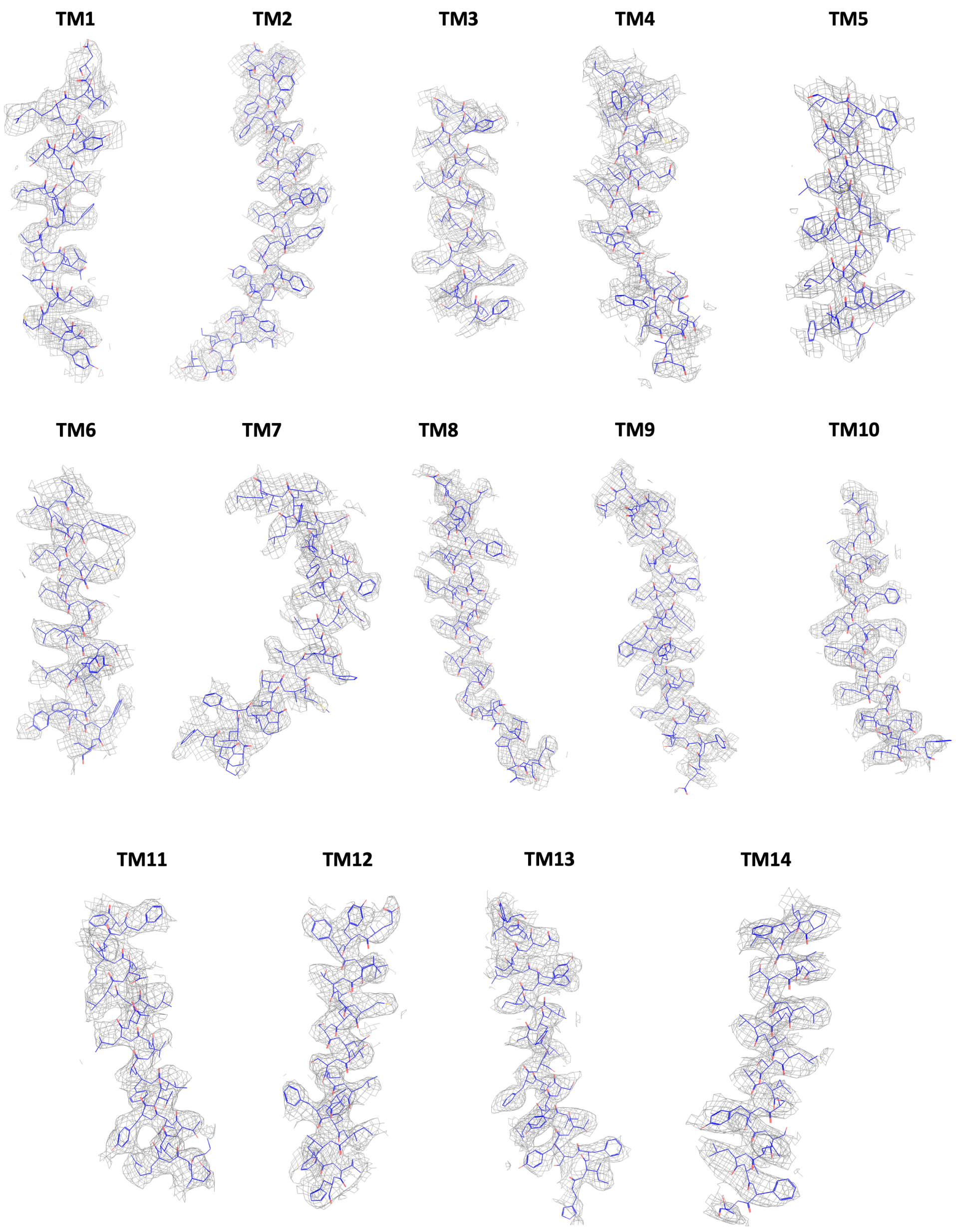

Supplementary Figure 4. Cryo-EM density of the refined TacF structure. The TacF model of TM1 through 14 is shown using line representation. Cryo-EM volume density is represented as a grey mesh.

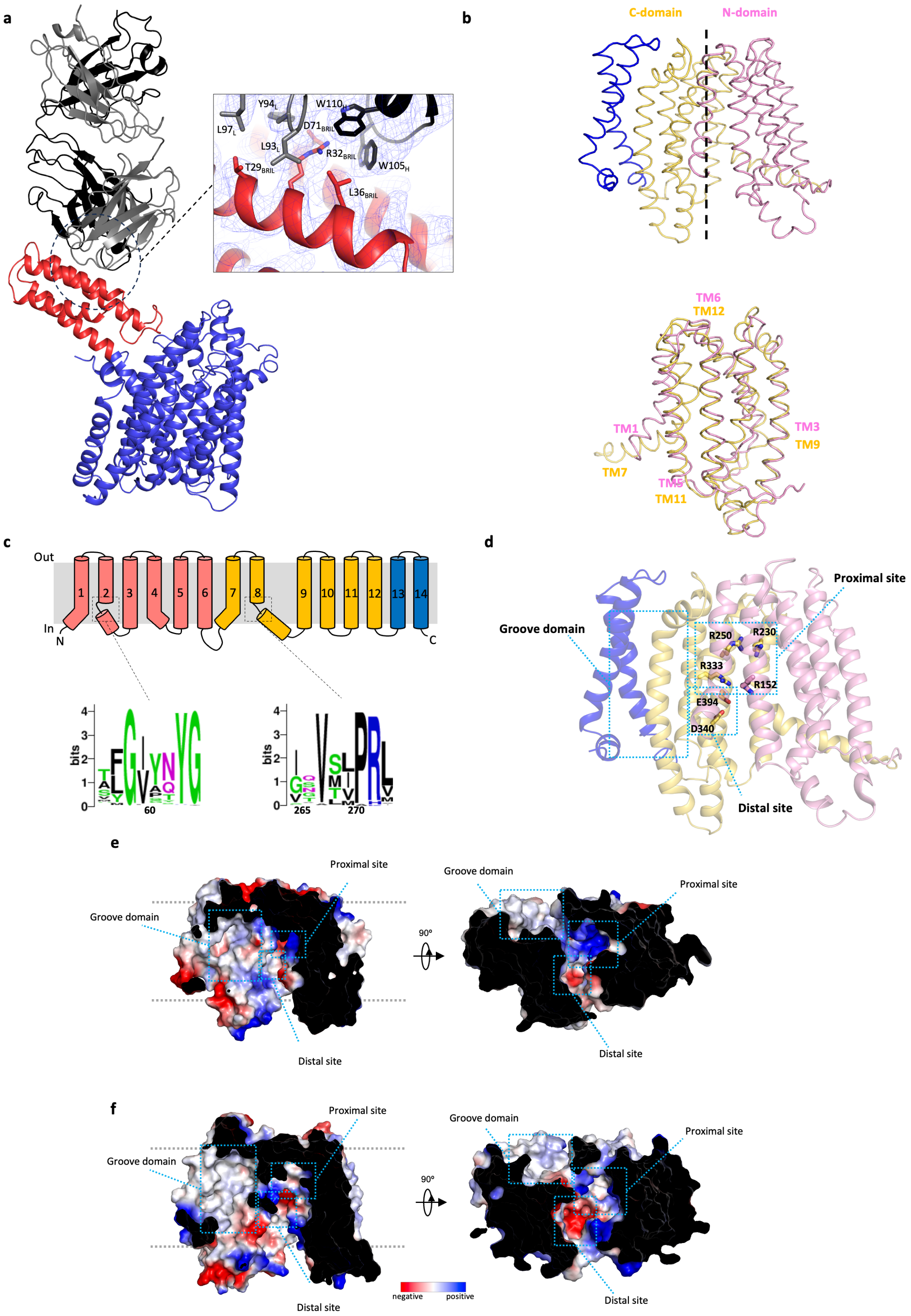

a
Y94L
W110H
L97L
D71BRIL
L93L
R32BRIL
W105H
T29BRIL
L36BRIL
b
C-domain
N-domain
TM6
TM12
TM1
TM3
TM9
TM7
TM5
TM11
c
Out
In
N
C
1
2
3
4
5
6
7
8
9
10
11
12
13
14
bits
60
265
270
d
Proximal site
Groove domain
R250
R230
R333
R152
E394
D340
Distal site
e
Proximal site
Groove domain
90°
Distal site
f
negative
positive

Supplementary Figure 5. Analysis of the TacF structure and topology. **A**. The structure of $BRIL_9$-TacF (red and blue, respectively) in complex with BAG2 Fab (Grey) is shown. *Inlet* shows the interactions involved in the binding of BAG2 to the BRIL fragment. The cryo-EM map is displayed as a blue mesh. **B**. Pseudosymmetry of the N- and C-terminal domains. *Bottom*, N-terminal (pink ribbons) and C-terminal (yellow ribbons) are superimposed. **C**. Topology of TacF based on the cryo-EM structure and sequence logos analysis of unwound segments observed in TM2 and TM8. **D**. Residues contributing to the positive surface of the proximal site and the negative charge of the distal site. **E** and **F**. Side and cytoplasmic views of a surface electrostatic potential representation of MurJ (PDB ID: 5T77) (**E**) and an AlphaFold model of Rft1 (**F**), showing the locations of the proximal and distal sites, as well as the groove domain.

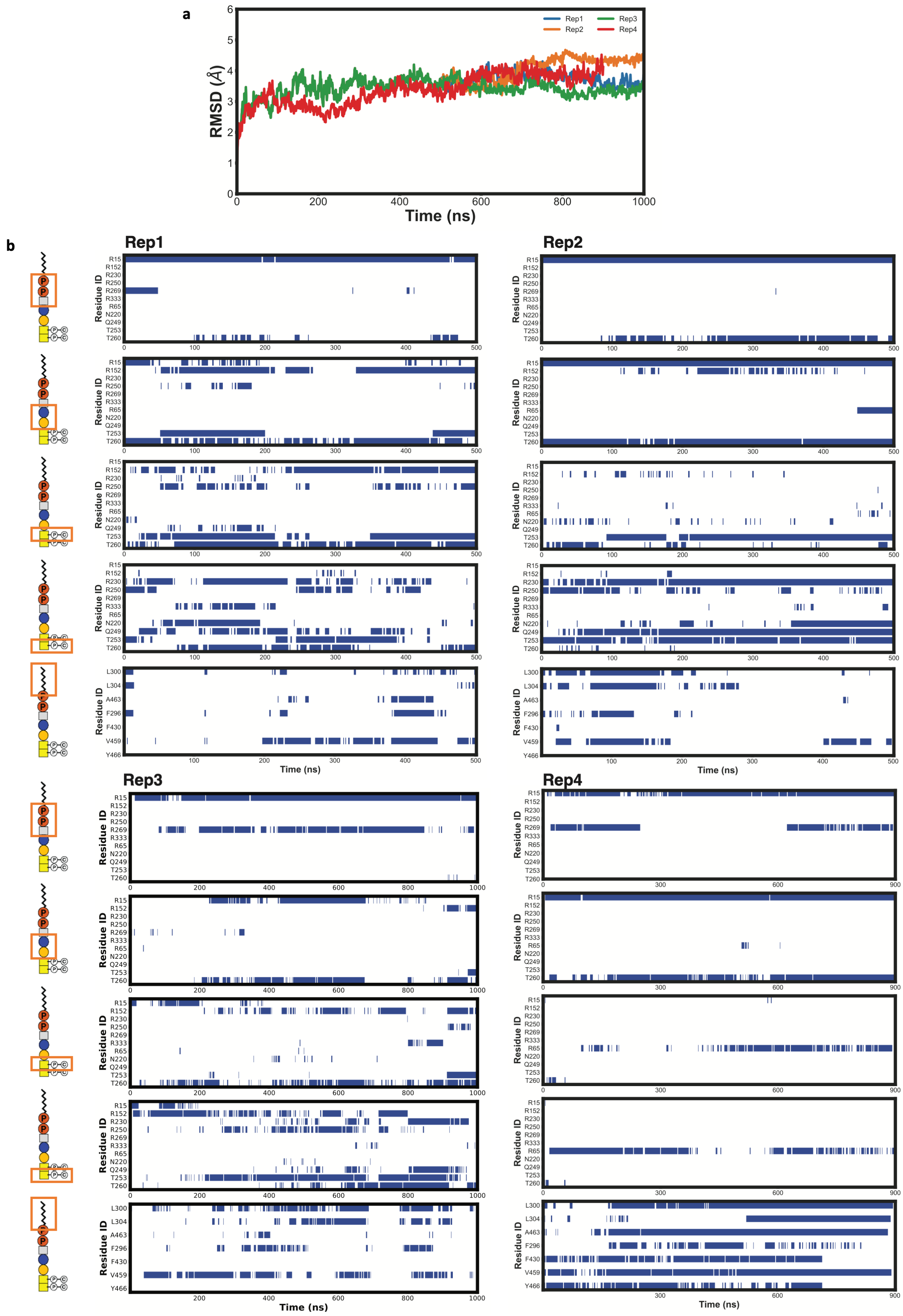

a
RMSD (Å)
Time (ns)
Rep1
Rep2
Rep3
Rep4
b
Rep1
Rep2
Rep3
Rep4
Residue ID
R15
R152
R230
R250
R269
R333
R65
N220
Q249
T253
T260
L300
L304
A463
F296
F430
V459
Y466
Time (ns)

c

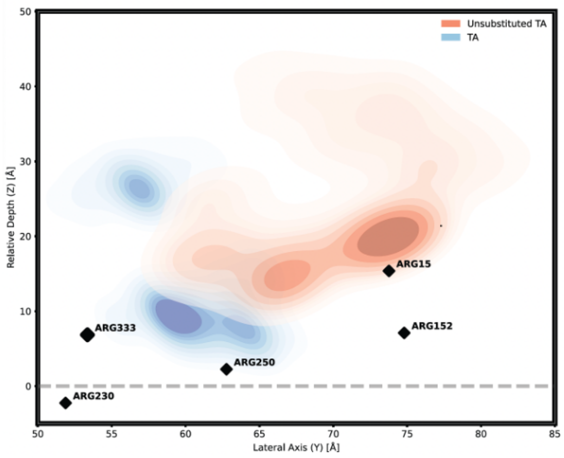

d

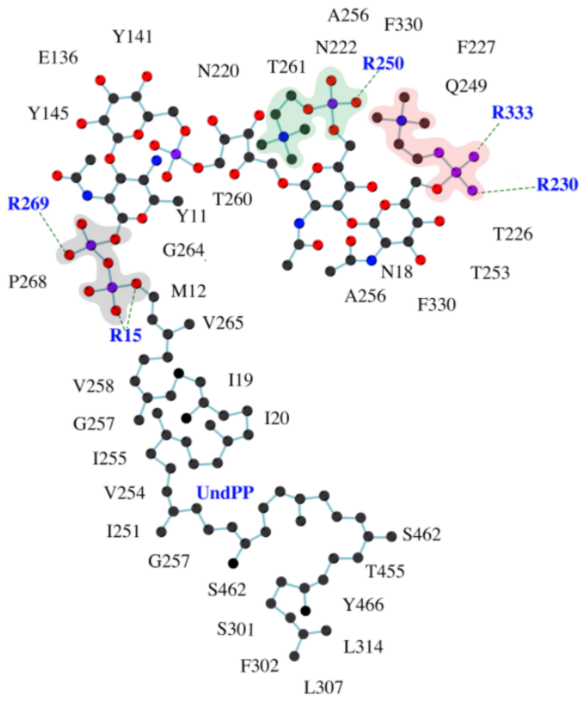

Supplementary Figure 6. MD simulations of TacF and interactions with teichoic acid. **A**. The root-mean-square-deviation (RMSD) of TacF starting from the equilibration structure during the different simulation replicates. **B**. The time evolution of the interaction of teichoic acid with residues in the binding cavity. Interactions between teichoic acid and an amino acid were considered if at least one pair of their non-hydrogen atoms was within 4 Å of each other. The interactions are shown for the five different parts of the teichoic acid separately (chemical structure representation of teichoic acid as shown in **Fig. 1A**). **C**. Kernel density estimation (KDE) plot showing the spatial distribution of the phosphocholine groups' center-of-mass (CoM) for the modified (blue) and unmodified teichoic acid (red) ligands across all MD replicas (for the unmodified teichoic acid, the remaining hydroxyl group is used as the CoM). The vertical axis represents the relative depth (Relative distance Z), normalized to a baseline defined by the binding cavity "floor" (residues R230 and R250), while the horizontal axis (Lateral axis Y) represents lateral movement within the pocket. Black diamonds indicate the relative positions of key architectural residues (e.g., C-alpha atoms of residues R15, R152, R230, R250, and R333) used as landmarks. The contrast between shaded and contoured regions highlights differences in pocket penetration and stable binding orientations between the two ligands. **D**. 2D representation of interactions between teichoic acid and TacF as seen in MD simulations (**Figure**

3A). Hydrogen bonds (green) indicate contacts with the lipid tail (UndPP), as well as interactions with the headgroup, including the phosphate moiety (shaded gray). The two phosphocholine groups (green and red), attached to the GlcNAc units, and the surrounding protein residues participating in these interactions are highlighted.

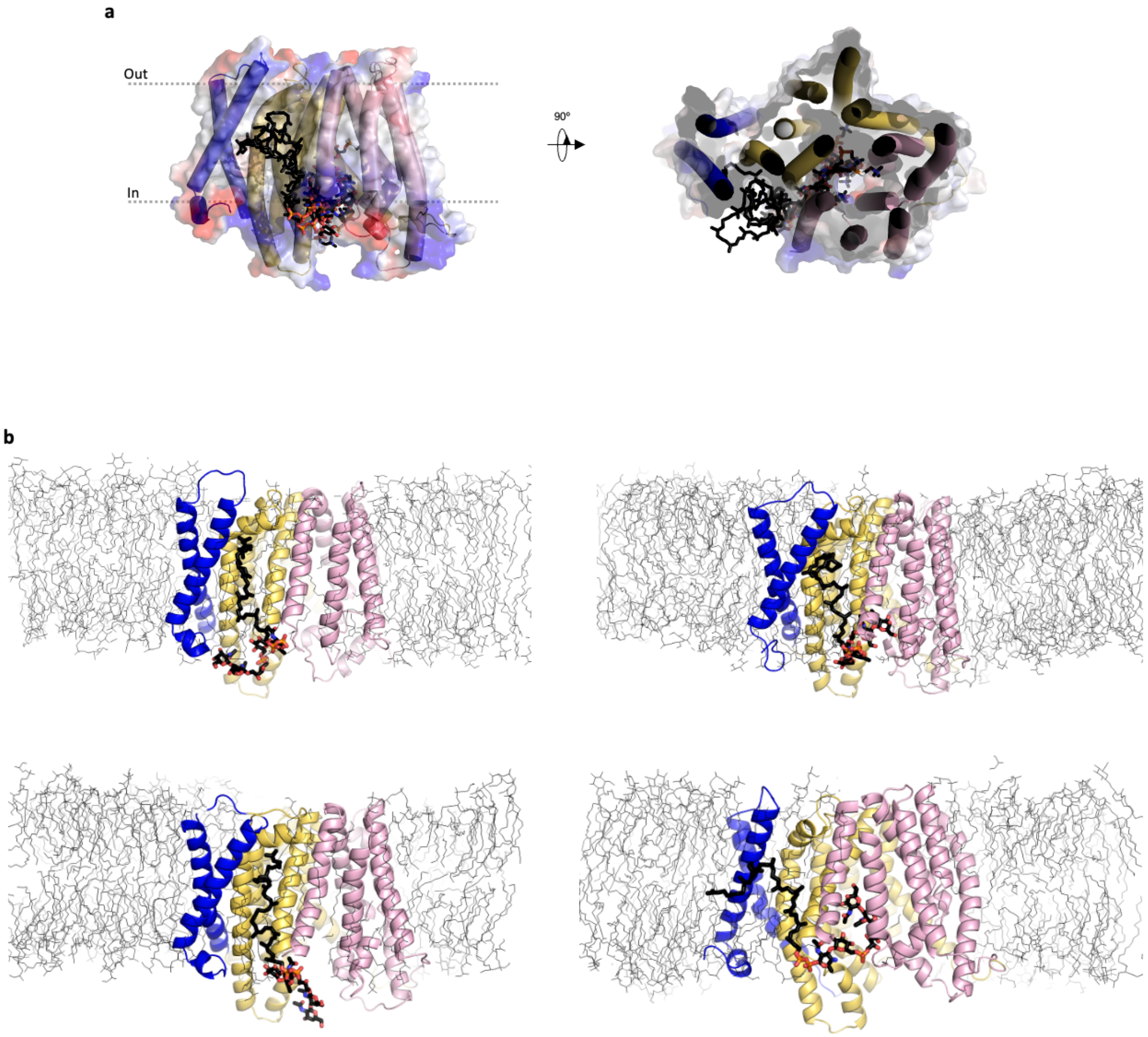

Supplementary Figure 7. Analysis of teichoic acid undecaprenyl tail and repeating unit in MD simulations. **A**. Representative snapshots of the major clusters occupied by the undecaprenyl tail in the MD simulations shown in **Figure 3A**. **B**. Snapshots from four independent MD simulations of TacF embedded in a heterogeneous bilayer, with a teichoic acid molecule that does not carry the phosphocholine modifications.

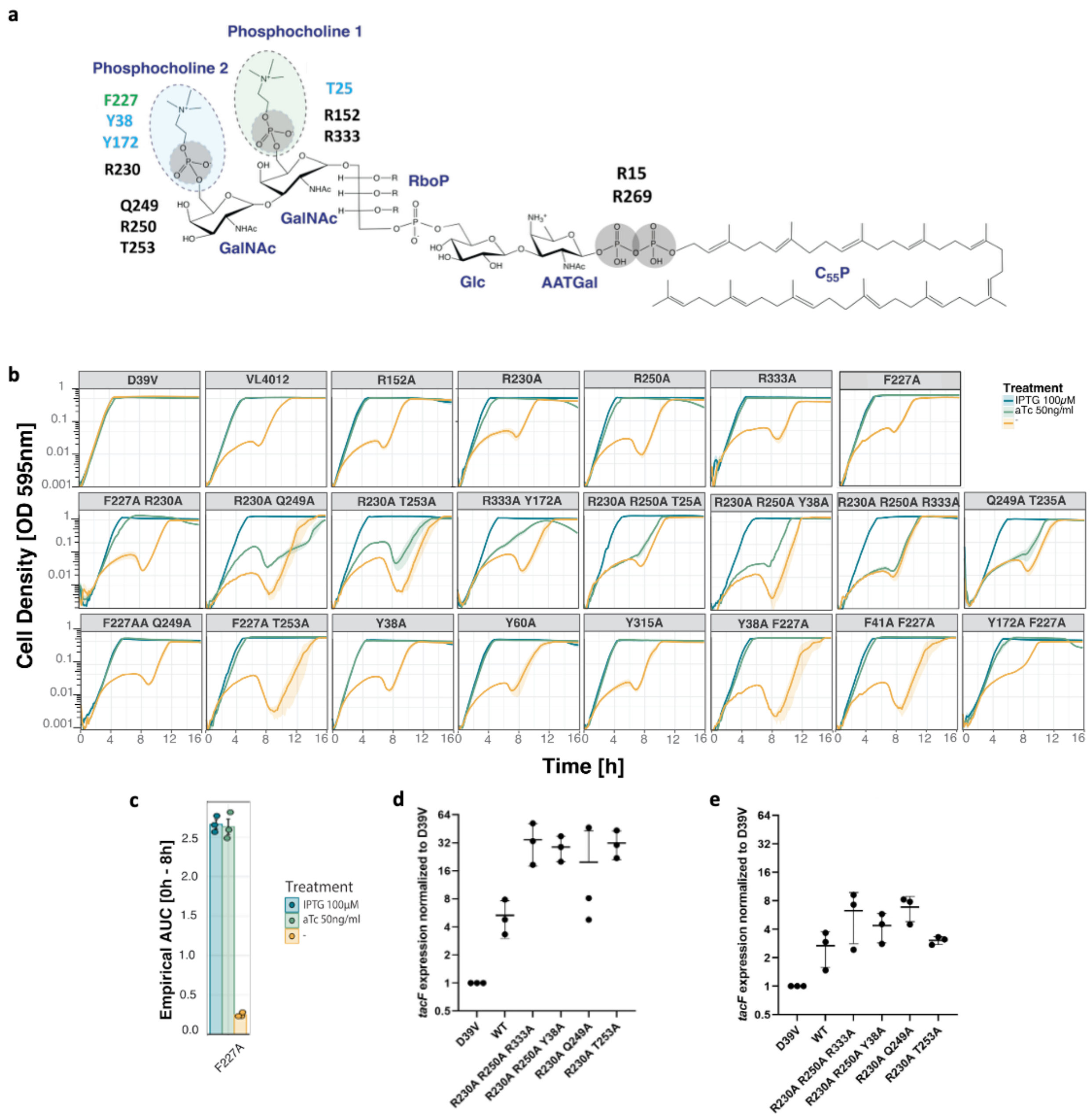

Supplementary Figure 8. A. row. **B**. Growth curves of *S. pneumoniae* strains carrying variants of TacF. 16h growth curves of *S. pneumoniae* D39V strains carrying the double expression system for the *tacF* wild type allele (VL4012) or *tacF* variants are shown. Cultures were grown in media supplemented with IPTG (blue), aTc (green), or no inducer (yellow). *S. pneumoniae* D39V growth curve indicates growth of the unmodified D39V strain. **C**. Bar plot showing the growth phenotype of *S. pneumoniae* strain carrying the double expression system for *tacF* variant F227A (see growth curve in **B**). **D** and **E**. Relative expression of *tacF* was quantified by qPCR and normalized to the

D39V parental strain. Expression levels are shown for D39V and the indicated variant strains. Each dot represents an individual biological replicate, and horizontal lines indicate the mean ± s.d. Data are plotted on a logarithmic scale. Bacterial strains were grown in fresh C+Y medium (pH 6.8) containing 1 mM IPTG or 50 ng/mL anhydrotetracycline (aTc) at 37 °C under 5% CO_2 to mid-log phase (OD_{600} = 0.3), followed by RNA extraction. Panels indicate induction with (**D**) IPTG or (**E**) aTc. Primer amplification efficiency was assessed and confirmed to approximate two-fold amplification per PCR cycle. n = 3 biological replicates; n = 3 technical replicates, for all experiments. Source data are provided as a Source Data file. Error bars shown are presented as mean values +/- SD (standard deviation).

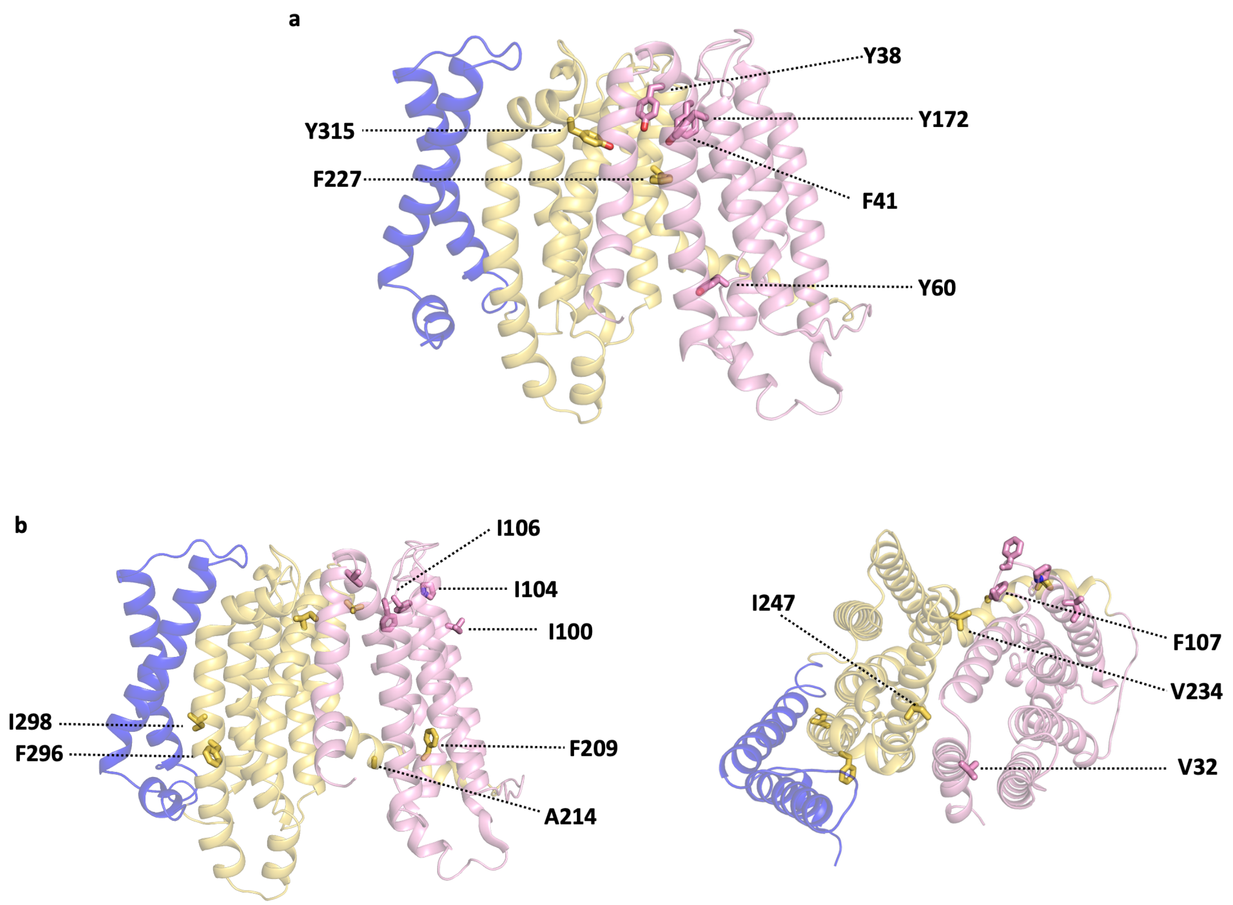

Supplementary Figure 9. Analysis of the TacF structure. **A**. Aromatic residues located in the central cavity of TacF. **B**. Residues that, when mutated, have been previously reported to induce promiscuous activity of TacF towards teichoic acids lacking phosphocholine[23,48,50,93].

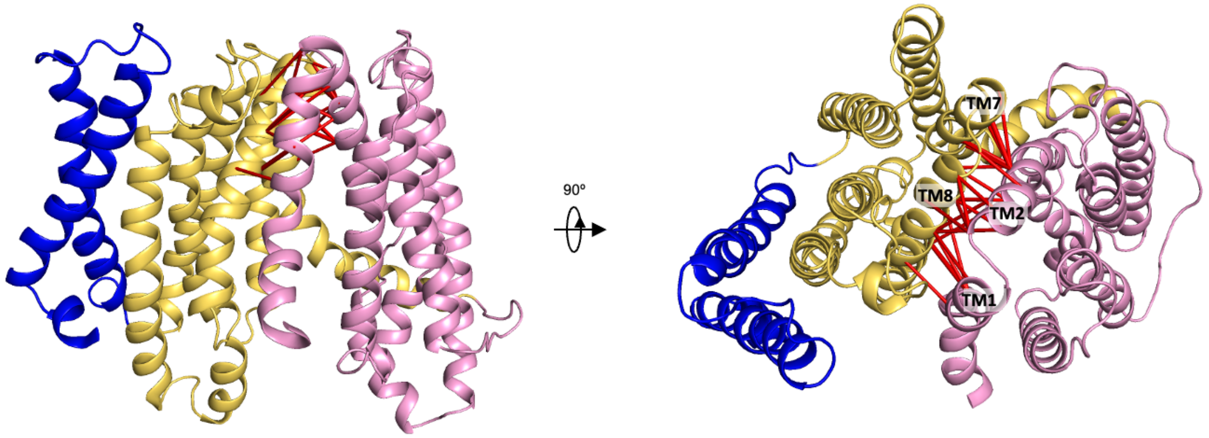

Supplementary Figure 10. Evolutionary coupling analysis of TacF. Coevolving pairs (red lines) with short inter-residue distances on the extracellular side of the cryo-EM TacF structure.

Supplementary Table 1. Primers used to generate the BRIL-TacF constructs

Primer	Sequence	T_m (°C)
$BRIL_5$-TacF For	5'- CAT ATA TCC AGA AGT ATC TTA AAC TGA ACG CCC TGA GCT ATA TGG GTA TTC-3’	68.7
$BRIL_7$-TacF For	5'- CAT ATA TCC AGA AGT ATC TTA ACG CCC TGA GCT ATA TGG GTA TTC-3’	66.9
$BRIL_8$-TacF For	5'- CAT ATA TCC AGA AGT ATC TTG CCC TGA GCT ATA TGG GTA TTC -3’	65.9
$BRIL_9$-TacF For	5'- CAT ATA TCC AGA AGT ATC TTC TGA GCT ATA TGG GTA TTC - 3’	62.7
$BRIL_{10}$-TacF For	5'- CAT ATA TCC AGA AGT ATC TTA GCT ATA TGG GTA TTC - 3’	59.7
BRIL-TacF Rev	5'- AAG ATA CTT CTG GAT ATA TGC ATT GCG GGT-3’	60.6
tacF RT PCR For	TCGTGGCTCCTGAATACTACT	63.3
tacF RT PCR Rev	AGCTAAAGATATGTCCCAAGTGG	63.1
gyrA RT PCR For	GACCCCAGACAAACCCCATA	64.6
gyrA RT PCR Rev	CACCATTGAGCCATACGGAC	63.7

Supplementary Table 2. Annotated LicA and LicD proteins from bacterial species from cluster I

	Accession code (Uniprot)	
**Species**	**LicA proteins**	**LicD proteins**
Streptococcus pneumoniae	Q8DPI4	A0A4J2CFT5
Streptococcus oralis	A0A1X1GPW3	A0A7T3DYT6
Streptococcus mitis	A0A7G1ITW7	A0A1X1KJM6
Streptococcus pseudoneumoniae	A0ABX9P8Y2	A0ABX9PAY6
Streptococcus infantis	E8JZW5	A0A0F3HB41
Streptococcus peroris	E8KAY8	E8KAG0
Streptococcus australis	A0A4V0BPR9	A0A4V0BMV8
Streptococcus gwangjuense	A0A387B3N7	A0A387B4I9
Streptococcus symci	A0A501PFD0	A0A501PFW3
Streptococcus halitosis	A0A426FYT2	A0A3R8LR66
Abiotrophia defectiva	W1Q510	W1Q2F3
Clostridium cadaveris	A0A1I2LYP8	A0A316MBX8
Clostridium paraputrificum	A0A1B8RP39	A0A6N3DGP0
Gemella bergeri	U2QU85	U2QID1
Gemella haemolysans	A0A134A6Z6	A0ABX6KJ87
Gemella morbillorum	A0AAP9KT11	93206785 (*ncbi gene ID*)
Gemella sanguinis	A0A2N6SEC6	A0ABX6FG15
Granulicatella balaenopterae	A0A1H9I4C8	A0A1H9NE57
Granulicatella elegans	D0BKR4	D0BJJ9
Parvimonas micra	A0A0B4S3M2	A0AAX3K5L5
Solibaculum mannosilyticum	*Not annotated*	A0A7I8CZ85

Supplementary Table 3. Molecular Dynamics simulation performed in this work

System	Time (µs)	System Size (No. of atoms)	System size (nm)
TacF (IF) with TA[1]	3.9 (0.5 × 4, 1.0, 0.9)	151224	12.0 × 12.0 × 10.3
TacF (IF) with TA without phosphocholines[1]	2.0 (0.5 × 4)	151180	12.0 × 12.0 × 10.2
TacF (OF) with TA[2]	0.9 (0.3 × 3)	142474	11.9 × 11.9 × 9.9

[1]Ligand was positioned near the lateral opening between TM1 and TM8, with the head group pointing towards the binding cavity. [2]The headgroup of the teichoic acid was docked first into the binding cavity, and then the undecaprenyl tail was added to the ligand.

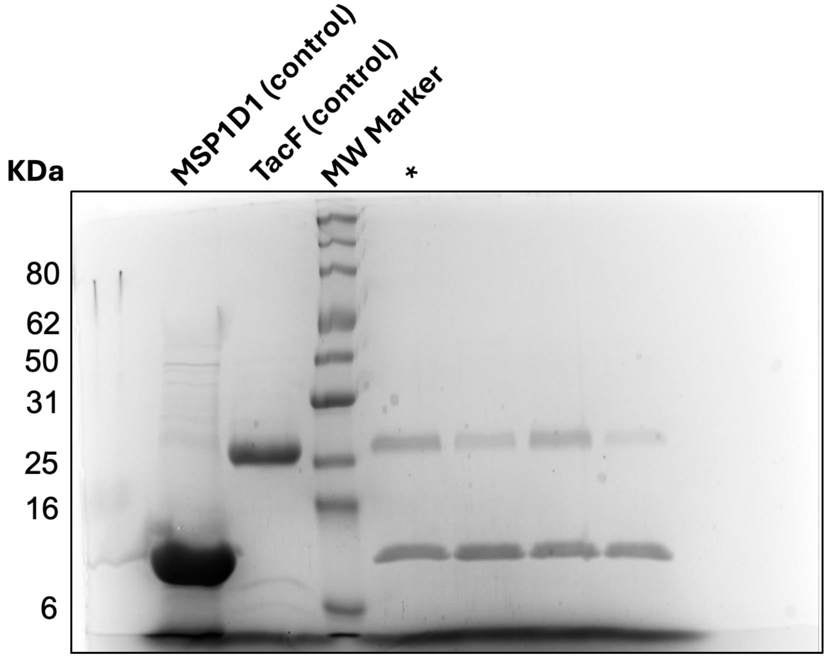

Source Data Supplementary Figure 1A.

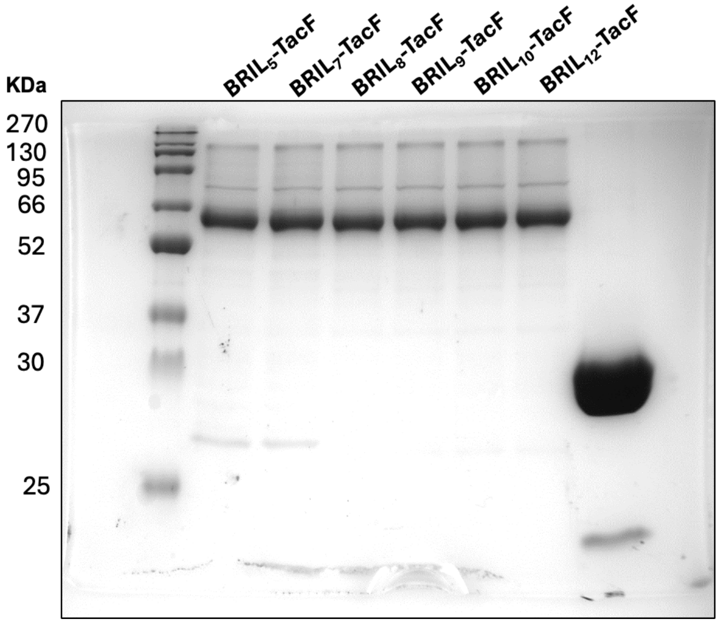

Source Data Supplementary Figure 2B.

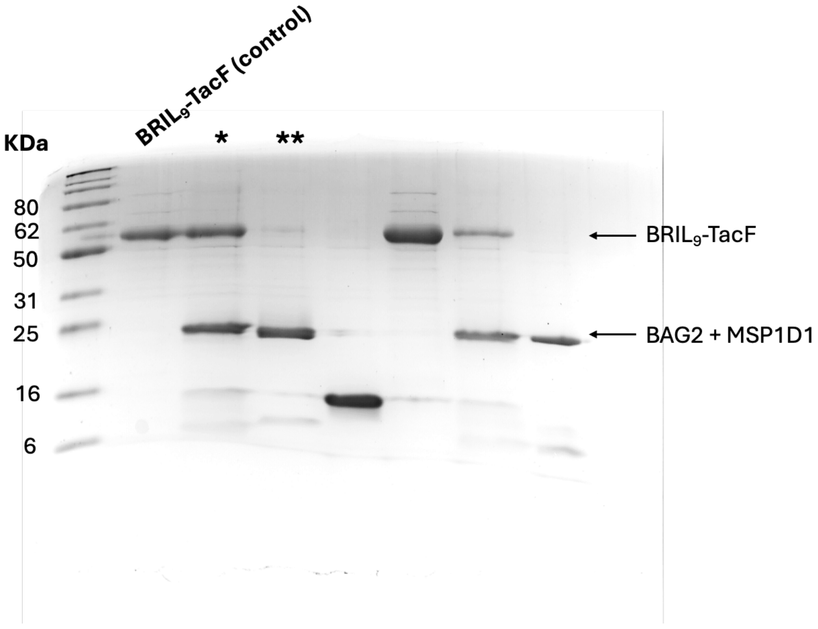

Source Data Supplementary Figure 3A.

**Reliability and reproducibility checklist for molecular dynamics simulations**<br>***All boxes must be marked YES by acceptance unless an N/A option is available**	**Yes**	**N/A**	**Response**<br>**(Please state where this information can be found in the text)**
**1. Convergence of simulations and analysis**			
1a. Is an evaluation presented in the text to show that the property being measured has equilibrated in the simulations<br>(*e.g.* time-course analysis)?	☒		Suppl Fig. 6
1b. Then, is it described in the text how simulations are split into equilibration and production runs and how much data were analyzed from production runs?	☒		Method section: MD simulations
1c. Are there at least 3 simulations per simulation condition with statistical analysis?	☒		Method section and Suppl. Table 3
1d. Is evidence provided in the text that the simulation results presented are independent of initial configuration?	☒		Suppl Fig. 6 and 7
**2. Connection to experiments**			
2a. Are calculations provided that can connect to experiments (*e.g.* loss or gain in function from mutagenesis, binding assays, NMR chemical shifts, J-couplings, SAXS curves, interaction distances or FRET distances, structure factors, diffusion coefficients, bulk modulus and other mechanical properties, *etc.*)?	☒		Results section (subsection: Functional characterization of residues involved in teichoic acid recognition)
**3. Method choice**			
3a. Is it described in the text what force field and water model are used and why?	☒		Method section: subsection: MD simulations
3b. Do simulations contain membranes, membrane proteins, intrinsically disordered proteins, glycans, nucleic acids, polymers, or cryptic ligand binding?	☒	☐	Response not needed if **N/A**
If 3b is **YES**, are enhanced sampling methods used?	☐	☒	Response not needed if **N/A**
If enhanced sampling methods are used, are the convergence criteria clearly stated?	☐		
If 3b is **YES**, is it explained in the text why or why not enhanced sampling methods are used?	☒		Results section (subsection: Functional characterization of residues involved in teichoic acid recognition)
**4. Code and reproducibility**			
4a. Is a table provided describing the system setup, such as simulation box dimensions, total number of atoms, total number of water molecules, salt concentration, lipid composition (number of molecules and type)?	☒		Method section and Suppl. Table 3
4b. Is it described in the text what simulation and analysis software and which versions are used?	☒		Method section
4c. Are initial coordinate and simulation input files and a coordinate file of the final output provided as supplementary files or in a public repository?	☒		Data availability section<br>https://doi.org/10.5281/zenodo.17151412

4d. Is there custom code or custom force field parameters?		☐	☒	Response not needed if **N/A**
	If **YES**, are they provided as supplementary profiles or in a public repository?	☐		
