# Supplementary material for: Mechanistic basis of teichoic acid transport by a gatekeeper flippase
Source: Nat Commun. 2026 May 25;17:6809. doi: 10.1038/s41467-026-73616-w (PMC13385700; doi:10.1038/s41467-026-73616-w)
Supplement: Supplementary file 2 — Description of Additional Supplementary Files [file 41467_2026_73616_MOESM2_ESM.pdf]

Description of Additional Supplementary Files

File name: Supplementary Movie 1

Description: Molecular Dynamics simulations showing the interaction of a teichoic acid molecule with the inward-facing state of TacF.
