# Supplementary material for: Mechanistic basis of teichoic acid transport by a gatekeeper flippase
Source: Nat Commun. 2026 May 25;17:6809. doi: 10.1038/s41467-026-73616-w (PMC13385700; doi:10.1038/s41467-026-73616-w)
Supplement: Supplementary file 5 — Transparent Peer Review file [file 41467_2026_73616_MOESM5_ESM.pdf]

nature portfolio

Peer Review File

Mechanistic basis of teichoic acid transport by a gatekeeper flippase

Corresponding Author: Professor Camilo Perez

This file contains all reviewer reports in order by version, followed by all author rebuttals in order by version.

Version 0:

Reviewer comments:

Reviewer #1

(Remarks to the Author)
The manuscript by Cebrero et al. describes the use of single-particle cryo-EM to obtain the structure of TacF, a lipid flippase in Streptococcus pneumoniae involved in teichoic acid transport within the bacterial membrane. Overall, the study is well written, and the figures are organized and clear. The results presented are robust, particularly the map presented is of high quality and the use of BRIL as a fiduciary marker is useful for others to apply to their own work. However, we have several comments which we believe could improve the paper and may require additional experimental results.

The authors contend that up to now, our understanding of MOP transporters has been limited to the lipid flippase MurJ which is primarily involved in peptidoglycan synthesis in Gram-negative bacteria. This is largely true as there are now several available structures of MurJ in the outward and inward conformations as well as MD simulations of the MurJ-Lipid II complex. In fact, the present study shares many similarities in figures to the original publication presenting the structure of MurJ from E. coli (https://www.pnas.org/doi/10.1073/pnas.1802192115). So, while we agree TacF is not 1:1 to MurJ, there stands numerous similarities which the authors frequently make mention of. While the use of sequence and conservation analysis reinforces that TacF may be a part of a different cluster than MurJ, observing Figure 5e makes it clear how similar the enzymes are. As it stands, the data presented while of high quality are somewhat incremental to the field and the claim that this process remains unknown in Gram-positive species is a bit misleading when the mechanism is likely to be near identical to MurJ.

We believe that to substantially build upon our knowledge from MurJ and relate it to homologs in Gram-positive bacteria, a substrate-bound state with a partial lipid (such as UndPP which is commercially available) or determination of an outward-facing state could help our understanding of how this may differ from a MurJ mechanism. As such, MurJ has yet to be solved with bound-substrate, so this could also inform on substrate position and residues that may be of importance. Short of this, it would be great if the authors could provide an argument why a substrate-bound state was not explored/possible (or necessary; Why was a undecaprenyl derivative not added to the nanodisc incorporation?) or could perform a set of biased, steered molecular dynamics simulations which could simulate an inward to outward state transition. We realize that asking for an additional structure is a major undertaking, but presently the proposed mechanism (which is where the interest lies) is inferred from MD simulations, comparisons to MurJ, and sequence analysis rather than solid structural data, and as such this objectively beautiful and solid work falls a little short in the scale of enthusiasm.

Minor comments:
Is it necessary to present all of the mass photometry and thermal stability data for the BRIL complexes in Main Figure 1? This may be better suited for an Extended Data Figure rather than a main. Perhaps present the data for the BRIL complex chosen and reserve the remaining constructs for the Supplementary. This would make the structure the primary focus of the figure, which is the most substantial result of the study.
Lines 45-48 – could use better clarity, sentence structure is confusing
Throughout check for capitalization of techniques particularly cryo-EM and molecular dynamics.

Reviewer #2

(Remarks to the Author)

I co-reviewed this manuscript with one of the reviewers who provided the listed reports. This is part of the Nature Communications initiative to facilitate training in peer review and to provide appropriate recognition for Early Career Researchers who co-review manuscripts.

Reviewer #3

(Remarks to the Author)
This manuscript describes the cryo EM analysis of TacF, a secondary transporter that mediates the translocation of a lipoteichoic acid precursor across the plasma membrane in gram-positive bacteria. The work integrates structural analysis with computational co-evolution and molecular dynamics analysis and in vivo functional assays. The results are interesting and will likely move the field forward.
The biogenesis of teichoic acid and related glycoconjugates remains poorly understood on a molecular level. Because teichoic acid is essential for the growth of many pathogens, detailed insights into key steps of its biosynthesis hold promise for targeted drug design.
The manuscript is well written and the conclusions are supported by the experimental data. However, I have a few points that should be addressed.

Major points
Introduction:
Please clearly state where the phosphocholine that modifies teichoic acid comes from. Fig. 1A suggests that it is from CDP-chol but this should be clearly stated to avoid confusion. Further, when introducing MOP transporters, please also state that these are secondary transporters that couple transport to an electrochemical gradient. Is it known what the counter-substrate of TacF is?

Results, SSN analysis:
The description of the clusters I-V could be improved. Specifically, the authors state that cluster I contains TacFs that are known to transport phosphocholine-derivatized teichoic acid. However, it would be informative to analyze whether all TacFs in this cluster indeed originate from bacteria that also encode LicD1 and LicD2, the phosphocholine transferases. If there is indeed a specific phosphocholine recognition motif, as suggested, I would assume that those should sort into a separate cluster.

Results, undecaprenyl binding:
I think this section could also be better described. Based on the description and the figures, it is unclear what features of the groove confer specificity for the isoprenyl units. Is it shape complementarity or something else? Why would the groove prefer to interact with isoprenyl units rather than acyl chains from phospholipids? Those would be in excess, hence some features must exist that confer specificity. Also, Fig. 3A seems to suggest that the undecaprenyl moiety adopts different poses after the MD simulations. Therefore, what features of the undecaprenyl chain are specifically recognized in all four repeats and what interactions differ? I don't think Suppl. Fig. 6B suffices to address this point.

Fig. 4 and in vivo complementation:
If I am not mistaken, Fig. 4a shows that strain VL4012 is viable in the absence of IPTG or aTc. Without induction, the culture reaches wild type density after about 12 hours and is at about 50% after 8 hours. So, why is it that the 'empirical AUC' shown in the bar graph is almost zero without inducer?
The authors further discovered that single point mutations near the substrate binding site have little to no effect on viability but that double and triple mutations essentially abolish growth. While this is not surprising, it is important that the mutant transporters are properly expressed and inserted into the plasma membrane. Otherwise, the analysis may not report on transporter function, but rather on folding and membrane integration.

Minor points:

Abstract and throughout the text: The statement that TacF 'monitors' the phosphocholine content of teichoic acid suggest that mono- or unsubstituted substrates are not recognized and transported at all. Is this indeed the case? If so, this could be stated more clearly.

Line 125:
Nanodiscs were prepared using POPG and DAG, described as major S. pneumoniae membrane components. However, MD simulations used 40% POPG, 20% DAG, and 40% CL. Please clarify why cardiolipin (CL) was omitted from nanodiscs yet included in the simulations.

Line 150:
"TM1 and TM8, positioned…" – suggest revising to "TM1 and TM8, respectively, positioned…" for clarity.

Lines 332 through 339:
Please clarify: "Our findings …. aligns with studies on S. pneumoniae growth in media where choline is replaced with ethanolamine". What did this study show, growth or no growth, formation of teichoic acid, etc?

Line 355:
Typo 'mutations win the tacF gene…'

Fig. 1:
It seems that the TacF structures in panels e and f/g are shown in different orientations. The cytosolic side is pointing up in panel e and down in panels f/g. This is confusing. Also, please add an arrow to panel f that shows the rotation direction and label TM helices 1 and 8 in the structure shown to the left.

Sample assembly for cryo-EM:
The description of the BRIL–TacF:BAG2 complex lacks detail. Please include protein concentrations and incubation time prior to size-exclusion chromatography, which produced the cryo-EM sample.

Line 413 – Protein expression:
Please include details on induction conditions (e.g., culture OD at induction).

Figure 4:
The figure and its discussion would benefit from the following clarifications:
- additional details on the D39V and VL4012 strains
- larger, more legible labels
- clarify whether R15 and R269 were tested, and if not, why.

Overall, the figures are quite small, which makes it hard to read the labels without zooming in.

Reviewer #4

(Remarks to the Author)
The current work by Cebrero et al. presents the structure and functional analysis of the Streptococcus pneumoniae TacF, a key flippase that monitors the phosphocholine content of teichoic acids (TA), using Cryo-EM, in vivo complementation assays, and in-silico molecular dynamics simulations. Due to the difficulty of obtaining a high-resolution reconstruction map for wild-type TacF, the cryo EM structure determination involved a clever way to first create fusion constructs of TacF with a marker “BRIL” and BRILL-binding synthetic antibody fragment BAG2. Of the six computationally (AF3) guided TacF constructs, BRIL9-TacF was pursued further for structure elucidation in nanodiscs, which resulted in a 3.63 Å resolution structure, with TacF structure in its inward-facing conformation in 2.5 Å resolution. The subsequent functional assays, evolutionary analysis, and MD simulations identified conserved motifs involved in substrate recognition and flippase activity. These investigations allowed the authors to propose a mechanistic model of the substrate recognition, binding, and flipping of PC-derived TA.
This work improves our understanding of the structure and mechanism of action of Streptococcus pneumoniae flippase TacF. While lipid II flippase MurJ structures have been extensively characterized, the lack of TacF structures poses a significant knowledge gap, and this work with the inward-facing TacF will be of great significance for the wider scientific community. Assessment of the work shows that the experimentations have been robust for both experiments and in silico investigations. However, a few points need to be addressed.
1) Substrate-binding
Experiment: Was a TacF complexed with the substrate attempted for structure determination?
Computational: The authors describe the positioning of TA “near the lateral entrance” between TM1 and TM8, but it’s unclear whether the placement was done with molecular docking, or a randomized placement between TM1/TM8 with TA already pulled up, or a randomized placement firmly embedded in the lipid bilayer. The description of how the TA was initially placed is important to correctly interpret the TA-protein residue interactions in the context of the solved structure and the timescale of the simulations. The phosphate groups appear to be placed in the proximal cavity. Did the tail fit in the hydrophobic groove? While substrate-bound structures of TacF are likely to be different, the rationale for choosing the proximal site as the initial putative binding site is unclear, especially because of low sequence conservation at that site. Also, the time-dependent interaction map (Fig. S6) isn’t very helpful without showing the binding poses. By 1 microsecond, the TA chain may have different clusters with varying residence times, and representative structures showing the interactions will be helpful. Since there was no movie or trajectory provided, it was difficult to see exactly what was done.
2) Insights on substrate placement/binding as well as the interaction patterns in different but related systems may offer some guidance (e.g., https://www.nature.com/articles/s41467-023-37702-7/figures/2; https://www.nature.com/articles/s41467-025-58196-5).
3) Mutational studies of S. pneumoniae growth have been outlined in Figure 4. However, R15 and R269, which are suggested to play a key role in the initial recognition and interaction of TA, have not been considered. Wouldn’t that provide strong insights into the presented mechanistic model?
4) Please provide the rationale (or a reference) for choosing the specific lipid composition in the methods. Also, describe whether it was an asymmetric bilayer (with corresponding compositions in the upper and lower leaflets).
5) Also mention the number of replicas and production lengths in the Methods.

Reviewer #5

(Remarks to the Author)
This manuscript provides a cryo-EM structure of S. pneumoniae TacF in lipid nanodiscs. TacF has been proposed to bind and translocate (flip) undecaprenol-phosphate-bound repeat units of teichoic acid across the plasma membrane. In addition to the structure, the authors perform sequence similarity analyses, site directed mutagenesis and molecular dynamic simulations to address the selectivity of TacF toward phosphocholine-substituted substrate that represents the mature

building block of teichoic acid in several bacterial species. Aided by the detailed understanding of other flippases including MurJ and RFT1, a model is proposed in Figure 6 to describe substrate recognition and translocation across the membrane. It is interesting that the cryo-EM structure of TacF (without substrate) is that of the inward facing protein; such that the inward state is also the resting state prone to accepting the substrate. The authors also propose a common evolutionary origin between these proteins and distinct key features for phosphocholine containing substrates. Overall, the study is well conducted and informative.

Comments:
1. Sup Fig 2: some sort of labeling should be used to delineate the BRIL segment from TacF.
2. Figure 2: the authors analyze many sequences including some from Staphylococcus, Bacillus and Clostridia. This is a bit confusing. Most Staph/Bacillus strains share similar WTA and the ABC transporter complex TarGH is used to translocate fully polymerized WTA polymers. Are the authors comparing TacF to TarGH or rather to LtaA-like enzymes for the assembly of the LTA glycolipid anchor? Clostridia teichoic assembly pathways are not clearly delineated so it is difficult to understand from the cited references to what Clostridial proteins TacF is being compared. In general, references #23, 63-65, cited in this section do not particularly provide clarity. Perhaps, the authors are simply collecting all proteins of the MOP family retrievable from Gram positive genomes?
3. Line 249: add ‘Figure 4C’ at the end of the sentence.
4. Figure 4b: in this panel, it would be nice to display R152, F227, Y172, T25, Y38 and some of the other substitutions tested in panels c,d,e.
5. Figure 4/Suppl Figure 7: Residues selected for mutations should all be accounted for in the text describing these data. For example, the authors should explain how combinations of residues to generate double and triple mutations were selected. As another example, the authors do not show the effect of F227A alone only in combination with other substitutions. Does F227A behave like wild type?
6. Figure 5: here the authors compare 138,448 sequences. How do these sequences relate to those in Figure 2?
7. Figure 6: do the authors propose that the enzyme returns to the inward facing state spontaneously? Is the undecaprenol-intermediate simply pushed outside of the channel? Could the action of TarP/TarQ facilitate the return to the inward state? At this stage, speculations are still allowed.

Reviewer #6

(Remarks to the Author)
I co-reviewed this manuscript with one of the reviewers who provided the listed reports. This is part of the Nature Communications initiative to facilitate training in peer review and to provide appropriate recognition for Early Career Researchers who co-review manuscripts.

Version 1:

Reviewer comments:

Reviewer #1

(Remarks to the Author)
The authors have addressed all of our main comments, and overall this is a greatly improved manuscript. We have no further comments if not that we still contend that Main Figure 1 would be far improved with less BRIL complex data to better highlight the protein structure results. As it stands, the figure is too large and the text is small.

Reviewer #2

(Remarks to the Author)
I co-reviewed this manuscript with one of the reviewers who provided the listed reports. This is part of the Nature Communications initiative to facilitate training in peer review and to provide appropriate recognition for Early Career Researchers who co-review manuscripts.

Reviewer #3

(Remarks to the Author)
The authors have addressed all of my comments to the best of their ability. I have no further comments.

Reviewer #4

(Remarks to the Author)
The revised manuscript by Cebrero et al. addressed the concerns pointed out in the initial round of review. Here are some minor comments about the modeling of the outward-facing conformation of TacF bound to teichoic acid (TA).

The authors used AF2 to model the outward-facing conformation of TacF and performed molecular docking of TA. The fragment docking approach taken by the authors seems to be appropriate, but the following are the comments/suggestions on the reconstruction of the molecule post-docking:

1. Since Glide SP only allows the ligand to be fully flexible and not the protein, manual placement of the TA tail to its polar headgroup post docking may need a short minimization to fully allow the TA molecule to be accommodated in the protein and account for side chain movements within the binding site that would otherwise not be accounted for in SP mode. Perhaps performing docking of the TA tail in the vicinity of the polar head group using the induced fit mode would avoid piecewise reconstruction.

2. An alternative, or an additional exercise, would be cofolding of TA and TacF using cofolding methods like Boltz-2 and Chai-2/Openfold3 that would enable AI-based prediction of not just the conformations but also the affinity of TA bound to TacF. Have the authors explored this?

Reviewer #5

(Remarks to the Author)
The authors have addressed my comments fully.

Reviewer #6

(Remarks to the Author)
I co-reviewed this manuscript with one of the reviewers who provided the listed reports. This is part of the Nature Communications initiative to facilitate training in peer review and to provide appropriate recognition for Early Career Researchers who co-review manuscripts.

Open Access This Peer Review File is licensed under a Creative Commons Attribution 4.0 International License, which permits use, sharing, adaptation, distribution and reproduction in any medium or format, as long as you give appropriate credit to the original author(s) and the source, provide a link to the Creative Commons license, and indicate if changes were made.
In cases where reviewers are anonymous, credit should be given to 'Anonymous Referee' and the source.
The images or other third party material in this Peer Review File are included in the article’s Creative Commons license, unless indicated otherwise in a credit line to the material. If material is not included in the article’s Creative Commons license and your intended use is not permitted by statutory regulation or exceeds the permitted use, you will need to obtain permission directly from the copyright holder.
To view a copy of this license, visit https://creativecommons.org/licenses/by/4.0/

Reviewer #1:

The manuscript by Cebrero et al. describes the use of single-particle cryo-EM to obtain the structure of TacF, a lipid flippase in Streptococcus pneumoniae involved in teichoic acid transport within the bacterial membrane. Overall, the study is well written, and the figures are organized and clear. The results presented are robust, particularly the map presented is of high quality and the use of BRIL as a fiduciary marker is useful for others to apply to their own work. However, we have several comments which we believe could improve the paper and may require additional experimental results.

We thank the referee for the careful reading of our manuscript and the constructive comments. Based on the reviewer's comments and concerns, we have revised our manuscript and hope these changes address the raised concerns.

The authors contend that up to now, our understanding of MOP transporters has been limited to the lipid flippase MurJ which is primarily involved in peptidoglycan synthesis in Gram-negative bacteria. This is largely true as there are now several available structures of MurJ in the outward and inward conformations as well as MD simulations of the MurJ-Lipid II complex. In fact, the present study shares many similarities in figures to the original publication presenting the structure of MurJ from E. coli (https://www.pnas.org/doi/10.1073/pnas.1802192115). So, while we agree TacF is not 1:1 to MurJ, there stands numerous similarities which the authors frequently make mention of. While the use of sequence and conservation analysis reinforces that TacF may be a part of a different cluster than MurJ, observing Figure 5e makes it clear how similar the enzymes are. As it stands, the data presented while of high quality are somewhat incremental to the field and the claim that this process remains unknown in Gram-positive species is a bit misleading when the mechanism is likely to be near identical to MurJ.

We thank the referee for their feedback. We have revised the abstract to include information about the structural similarity between MurJ and TacF. Please note that throughout the text, we carefully emphasize the similarities in the architecture and mechanism of MurJ and TacF to provide an honest report to the research community. We have updated the text to highlight that differences in the recognition mode of the headgroup (non-diphosphate) that exist when comparing the mechanisms of MurJ and TacF. Please see Lines 94, 225-227, and 403-407.

We also would like to emphasize the critical need to elucidate the mechanisms by which Gram-positive bacterial systems assemble cell wall biopolymers, since the surfaces of these bacteria differ markedly from those of Gram-negative bacteria. Thus, our study on TacF, being a protein central to teichoic acids synthesis, is highly relevant. We believe that elucidating how pathogens such as *S. pneumoniae* assemble their cell walls is a major undertaking with important implications for therapeutic interventions.

We believe that to substantially build upon our knowledge from MurJ and relate it to homologs in Gram-positive bacteria, a substrate-bound state with a partial lipid (such as UndPP which is commercially available) or determination of an outward-facing state could help our understanding of how this may differ from a MurJ mechanism. As such, MurJ has yet to be solved with bound-substrate, so this could also inform on substrate position and

residues that may be of importance. Short of this, it would be great if the authors could provide an argument why a substrate-bound state was not explored/possible (or necessary; Why was a undecaprenyl derivative not added to the nanodisc incorporation?) or could perform a set of biased, steered molecular dynamics simulations which could simulate an inward to outward state transition.
We realize that asking for an additional structure is a major undertaking, but presently the proposed mechanism (which is where the interest lies) is inferred from MD simulations, comparisons to MurJ, and sequence analysis rather than solid structural data, and as such this objectively beautiful and solid work falls a little short in the scale of enthusiasm.

We thank the reviewer for the insightful comment. Indeed, we attempted to obtain a high-resolution structure of a substrate-bound state, which could provide a clearer picture of teichoic acid binding by TacF. We invested significant effort in reconstituting the TacF-BRIL construct into nanodiscs, using lipid extracts from *S. pneumoniae* teichoic acids. Unfortunately, the reconstruction maps obtained using this sample have an average resolution of 8.5 Å, limiting analysis of the binding mode of teichoic acids to TacF (**See Figure below**). The volume obtained showed what we believe is a different conformational state, but due to the low resolution, it was not possible to reach a meaningful conclusion.

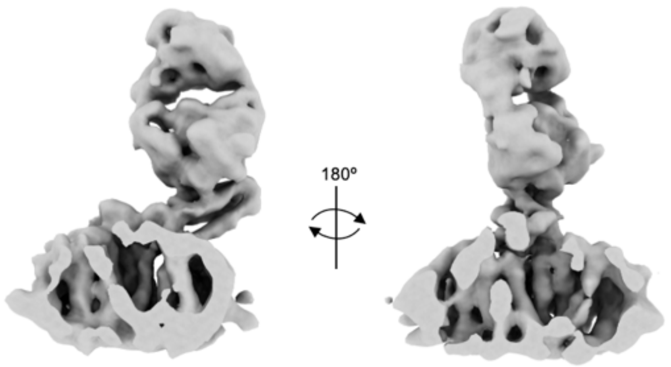

Figure. TacF reconstituted in nanodiscs inclusing teichoic acid lipid extracts. Sliced view of the 8.5Å reconstruction map of TacF:BRIL.

As mentioned by the reviewer, good alternative methods for obtaining substrate-bound structures involve using und-PP or other commercially available lipids. We agree with this; however, we have encountered multiple experimental hiccups, including a lack of high-resolution to resolve lipid features, and delays in conducting these experiments due to the rotation of the students involved in the project. These experiments are part of ongoing efforts, and we hope to report our findings soon.

As suggested by the reviewer, we have performed additional MD simulations that have now been incorporated into the manuscript. With these new results, we added analysis of teichoic acid binding to TacF, but now in an additional outward-facing conformational state. In this MD simulation, we continue to observe (i) strong interactions of the undecaprenyl tail with the hydrophobic groove; (ii) close interactions of the diphosphate group with positively charged residues, i.e. R15; (iii) interactions of the second GalNac unit with Q249 and R250; and (iv) interaction of the phosphate group of the second phosphocholine group with R230. In conclusion, most interactions established during teichoic acid recognition by inward-open TacF appear to be maintained during the transition to an outward-facing state. These results are now reported in Lines 244-252, 258-260, 384-388, and **Figures 3E-H**.

Minor comments:

Is it necessary to present all of the mass photometry and thermal stability data for the BRIL complexes in Main Figure 1? This may be better suited for an Extended Data Figure rather than a main. Perhaps present the data for the BRIL complex chosen and reserve the remaining constructs for the Supplementary. This would make the structure the primary focus of the figure, which is the most substantial result of the study.

We agree with the reviewer that BRIL serves as a useful fiducial marker and that its inclusion may be valuable for others seeking to apply similar approaches. For this reason, we have chosen not to modify Main Figure 1 and hope the reviewer will agree that it is best left in its current form.

Lines 45-48 – could use better clarity, sentence structure is confusing.

Thank you for pointing this out. We have changed the sentence to improve clarity.

Throughout check for capitalization of techniques particularly cryo-EM and molecular dynamics.

We have made the changes as suggested by the reviewer.

Reviewer #3:

This manuscript describes the cryo EM analysis of TacF, a secondary transporter that mediates the translocation of a lipoteichoic acid precursor across the plasma membrane in gram-positive bacteria. The work integrates structural analysis with computational co-evolution and molecular dynamics analysis and in vivo functional assays. The results are interesting and will likely move the field forward. The biogenesis of teichoic acid and related glycoconjugates remains poorly understood on a molecular level. Because teichoic acid is essential for the growth of many pathogens, detailed insights into key steps of its biosynthesis hold promise for targeted drug design.

We appreciate the reviewer's constructive feedback and comments on our manuscript. We have revised the manuscript to address the referee's concerns and suggestions.

The manuscript is well written and the conclusions are supported by the experimental data. However, I have a few points that should be addressed.

Major points:

Introduction:
Please clearly state where the phosphocholine that modifies teichoic acid comes from. Fig. 1A suggests that it is from CDP-chol but this should be clearly stated to avoid confusion.

We have provided additional information regarding the origin of the phosphocholine modification, as suggested by the reviewer. See Line 62-67.

Further, when introducing MOP transporters, please also state that these are secondary transporters that couple transport to an electrochemical gradient. Is it known what the counter-substrate of TacF is?

We thank the reviewer for the valuable and insightful comment. We have now included a statement about ion co-transport by MOP transporters and TacF. See Line 408-413.
Regarding the counter-substrate of TacF, there is currently no experimental evidence supporting the co-transport of ions or other molecules together with teichoic acids. Because teichoic acids are translocated downhill with respect to the electrochemical gradient, ion co-transport is not strictly required from an energetic standpoint. However, this observation alone is insufficient to rule out coupled transport. We therefore acknowledge that the identity of a potential counter-substrate remains unresolved and note that ongoing structural and functional studies in our laboratory are aimed at addressing this question.

Results, SSN analysis:
The description of the clusters I-V could be improved. Specifically, the authors state that cluster I contains TacFs that are known to transport phosphocholine-derivatized teichoic acid. However, it would be informative to analyze whether all TacFs in this cluster indeed originate from bacteria that also encode LicD1 and LicD2, the phosphocholine transferases. If there is indeed a specific phosphocholine recognition motif, as suggested, I would assume that those should sort into a separate cluster.

We thank the reviewer for raising this important point. As suggested, we have further analyzed the sequences in cluster-I, focusing on other genes that code for essential proteins in the phosphocholine modification pathway. Our search targeted LicA (the choline kinase that catalyzes the activation of choline to generate CDP-choline) and LicD (the enzyme that adds the phosphocholine group to teichoic acids) (see new **Suppl. Table 2**).

Since the activity of these proteins has not been confirmed across multiple bacterial species in cluster-I, we superimposed AlphaFold models of each protein with the *S. pneumoniae* LicA or LicD1/2 proteins to demonstrate their structural similarity. As shown in the **figures and tables below**, the superposition of C-alpha atoms results in low RMSD values, confirming that these proteins resemble *S. pneumoniae* LicA and LicD1/2, likely sharing the same function.

Overall, these results suggest that all TacF proteins grouped in cluster-I may be specialized flippases that transport phosphocholine-decorated teichoic acids, as suggested by similar distal and proximal site characteristics. However, further studies are needed to confirm this hypothesis. We have added new lines to summarize these findings. See Line 188-191.

RMSD for LicA proteins superposition

Species	RMSD (Å)
Streptococcus oralis	1.43
Streptococcus mitis	0.97
Streptococcus psdeudoneumoniae	1.55
Streptococcus infantis	1.51
Streptococcus peroris	1.54
Streptococcus australis	2.79
Streptococcus gwangjuense	1.33
Streptococcus symci	1.36
Streptococcus halitosis	1.50
Abiotrophia defectiva	1.93
Clostridium cadaveris	3.80
Clostridium paraputrificum	2.60
Gemella bergeri	1.45
Gemella haemolysans	1.45
Gemella morbillorum	1.84
Gemella sanguinis	2.35
Granulicatella balaenopterae	2.63
Granulicatella elegans	2.70
Parvimonas micra	2.72
Solibaculum mannosilyticum	Not annotated

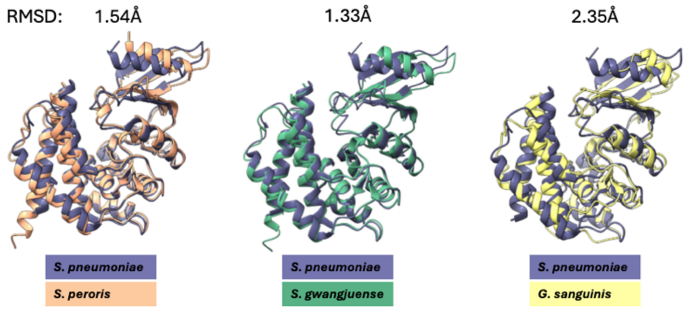

Figure. Superposition of AlphaFold-3 Models of LicA protein from diverse bacteria in cluster I Vs the crystal structure of LicA from *S. pneumoniae* (Uniprot accession code Q8DPI4).

RMSD for LicD proteins superposition

Species	RMSD (Å)
Streptococcus oralis	2.40
Streptococcus mitis	0.54
Streptococcus psdeudoneumoniae	2.32
Streptococcus infantis	1.05
Streptococcus peroris	0.52
Streptococcus australis	2.10
Streptococcus gwangjuense	1.09
Streptococcus symci	2.20
Streptococcus halitosis	2.48
Abiotrophia defectiva	2.22
Clostridium cadaveris	2.42
Clostridium paraputrificum	1.95
Gemella bergeri	2.20
Gemella haemolysans	2.14
Gemella morbillorum	2.40
Gemella sanguinis	0.96
Granulicatella balaenopterae	2.39
Granulicatella elegans	2.31
Parvimonas micra	2.06
Solibaculum mannosilyticum	2.70

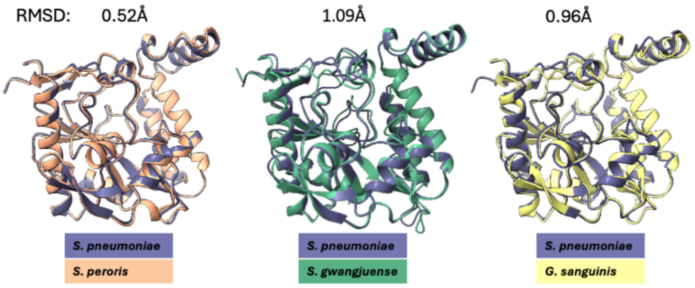

Figure. Superposition of AlphaFold-3 Models of LicD protein from diverse bacteria in cluster I Vs the AlphaFold-3 model of LicD1 from *S. pneumoniae* (Uniprot accession code A0A4J2CFT5).

Results, undecaprenyl binding:
I think this section could also be better described. Based on the description and the figures, it is unclear what features of the groove confer specificity for the isoprenyl units. Is it shape complementarity or something else? Why would the groove prefer to interact with isoprenyl units rather than acyl chains from phospholipids? Those would be in excess, hence some features must exist that confer specificity.
Also, Fig. 3A seems to suggest that the undecaprenyl moiety adopts different poses after the MD simulations. Therefore, what features of the undecaprenyl chain are specifically recognized in all four repeats and what interactions differ? I don't think Suppl. Fig. 6B suffices to address this point.

We thank the reviewer for raising this question. Previous studies have shown that undecaprenyl lipids adopt multiple conformations in membranes, including bent V- and L-shaped states[1,2,3], consistent with the conformational ensemble observed in our MD simulations. We propose that the hydrophobic groove preferentially accommodates the undecaprenyl tail due to (i) its geometry, which is compatible with both V- and L-shaped conformations (**Fig. 3A**), and (ii) the extensive hydrophobic contacts formed with the polyisoprenoid chain, which, due to its length, exceed those achievable with a single phospholipid acyl chain. This increased interaction surface is expected to prolong the residence time of the undecaprenyl tail within the groove. Consistent with this model, our simulations show that phospholipids occupy the groove for significantly shorter times than undecaprenyl lipids. Specifically, we calculated the mean residence time and the off-rate constant (k_{off}) of the undecaprenyl tail and three lipid species, POPG, DAG, and CL, in binding into the groove in all performed simulations of UT (total of 4.7 μs). The result shows a mean resident time of 75 ns for the undecaprenyl tail, whereas it was clearly lower for the lipid molecules (25, 28, and 17 ns for POPG, DAG, and CL, respectively). The k_{off} of the undecaprenyl

tail is also slower than that of the surrounding POPG, DAG, and CL molecules (5.78, 8.06, 11.7, and 12.4 μs^{-1} for undecaprenyl tail, POPG, DAG, and CL, respectively).
Additionally, we believe that the recognition of the undecaprenyl tail, the PP, and the repeating unit likely occurs almost simultaneously; therefore, we cannot rule out that the residence time of the isoprenyl tail in the groove is extended due to an allosteric effect from recognizing the other groups in the teichoic acid molecule.
In light of the reviewer's comments, we have revised the text accordingly. See Line 363-367.

1. Mulholland S, Turpin ER, Bonev BB, Hirst JD. Docking and molecular dynamics simulations of the ternary complex nisin2:lipid II. Sci Rep. 2016 Feb 18;6:21185. doi: 10.1038/srep21185. PMID: 26888784; PMCID: PMC4758073.
2. Chugunov A, Pyrkova D, Nolde D, Polyansky A, Pentkovsky V, Efremov R. Lipid-II forms potential "landing terrain" for lantibiotics in simulated bacterial membrane. Sci Rep. 2013;3:1678. doi: 10.1038/srep01678. PMID: 23588060; PMCID: PMC3627190.
3. Kuk ACY, Hao A, Guan Z, Lee SY. Visualizing conformation transitions of the Lipid II flippase MurJ. Nat Commun. 2019 Apr 15;10(1):1736. doi: 10.1038/s41467-019-09658-0. PMID: 30988294; PMCID: PMC6465408.

Fig. 4 and in vivo complementation:
If I am not mistaken, Fig. 4a shows that strain VL4012 is viable in the absence of IPTG or aTc. Without induction, the culture reaches wild type density after about 12 hours and is at about 50% after 8 hours. So, why is it that the 'empirical AUC' shown in the bar graph is almost zero without inducer?

We thank the reviewer for raising this important point. We would like to clarify that the cell density plots are displayed with a logarithmic scale on the Y-axis. This scaling may not have been immediately apparent to the reviewer. When the logarithmic scale is taken into account, the apparent discrepancy disappears. Accordingly, the empirically calculated AUC shown in the bar graph is indeed close to zero for VL4012 under non-inducing conditions.

The authors further discovered that single point mutations near the substrate binding site have little to no effect on viability but that double and triple mutations essentially abolish growth. While this is not surprising, it is important that the mutant transporters are properly expressed and inserted into the plasma membrane. Otherwise, the analysis may not report on transporter function, but rather on folding and membrane integration.

We thank the reviewer for pointing this out. We used quantitative PCR (qPCR) to compare transcript abundance across the double and triple mutants and confirm that any observed phenotypic differences were not due to reduced gene expression in the mutants. Quantitative PCR analysis revealed that *tacF* expression in all the strains was comparable to or exceeded that observed in the parental D39V strain under both IPTG- and aTc-induced conditions (**Supplementary Fig. 8B, D,E**). Across all strains tested, *tacF* transcript levels were consistently elevated relative to D39V, indicating that *tacF* is not downregulated in these backgrounds. These data suggest that the growth defects observed in the mutant strains in growth assays are unlikely to arise from reduced *tacF* expression and instead reflect altered functional dependence on membrane-associated processes. Furthermore, the elevated transcription is somewhat expected, as IPTG and aTc induce gene expression from lac- and Tet-regulated systems, which, in turn, elicit this effect in response to inducer concentration[1]. It is also reassuring that not all mutations generated by the same mutagenesis strategy cause growth

defects, since this would have cast doubt on the validity of the system. These results have now been included in **Supplementary Figure 8D,E** and mentioned in the text. See Line 284-286.

1. Stamsas, G.A. et al. CozEa and CozEb play overlapping and essential roles in controlling cell division in Staphylococcus aureus. Mol. Microbiol.109, 615–632 (2018).

We would like to point out that directly verifying protein synthesis and correct membrane insertion for all the mutants is highly challenging, technically demanding, and requires specialized instrumentation and the development of new protocols, such as quantitative mass spectrometry with heavy-isotope labeling, particularly for low-abundance membrane proteins. Given the current circumstances, we are unable to perform protein-level expression experiments at this time.

Minor points:

Abstract and throughout the text: The statement that TacF 'monitors' the phosphocholine content of teichoic acid suggest that mono- or unsubstituted substrates are not recognized and transported at all. Is this indeed the case? If so, this could be stated more clearly.

Thanks to the reviewer for raising this point. There is no evidence regarding the transport of mono-substituted substrates. We now present new MD simulations with an unsubstituted teichoic acid molecule. We observed that the unsubstituted teichoic acid does not enter into the binding site during 4×500 ns simulations, hinting that the unsubstituted teichoic acid might have a lower affinity for the binding site. We have modified the text to include these new results. See Line 228-236 and **Suppl. Fig. 7B**.

It is important to note that the lifetime of mono-ad unsubstituted teichoic acids in the membrane depends on the catalytic rates of LicD1 and LicD2. Currently, there is no detailed enzymatic analysis of these enzymes. However, based on the rates of enzymes that catalyze similar processes, one could hypothesize that the kcat for the modification of teichoic acids by LicD1 and LicD2 is in the range of 0.01–1 s^{-1}, which is well above the expected transport rate of a flippase. Future studies that measure the rates of these processes will shed further light on this question.

Line 125:
Nanodiscs were prepared using POPG and DAG, described as major *S. pneumoniae* membrane components. However, MD simulations used 40% POPG, 20% DAG, and 40% CL. Please clarify why cardiolipin (CL) was omitted from nanodiscs yet included in the simulations.

We thank the reviewer for pointing this out. We attempted to incorporate cardiolipin into our nanodisc preparations; however, under the conditions tested, the presence of cardiolipin consistently resulted in poor nanodisc assembly and sample heterogeneity, preventing the formation of stable and homogeneous preparations suitable for structural analyses. For this reason, cardiolipin was omitted from the final nanodisc preparations used for cryo-EM studies.

Line 150:
"TM1 and TM8, positioned…" – suggest revising to "TM1 and TM8, respectively, positioned…" for clarity.

We have implemented the changes recommended by the reviewer.

Lines 332 through 339:
Please clarify: "Our findings …. aligns with studies on S. pneumoniae growth in media where choline is replaced with ethanolamine". What did this study show, growth or no growth, formation of teichoic acid, etc?

The studies by Tomasz et al., 1968, López et al., 1982, and Ware et al., 2005 [1,2,3], showed that *S. pneumoniae* can grow in media where choline is replaced by ethanolamine, which is incorporated into wall and lipoteichoic acids in place of phosphocholine. However, such growth is accompanied by pronounced phenotypic alterations, including long-chain formation, impaired cell separation during division, and reduced autolysis, reflecting the loss of choline-dependent interactions with autolysins and other surface proteins (i.e. choline binding proteins). These findings indicate that while teichoic acid synthesis can proceed with ethanolamine, phosphocholine is critical for normal cell wall function. See line 435.

1. Tomasz A. Biological consequences of the replacement of choline by ethanolamine in the cell wall of Pneumococcus: chanin formation, loss of transformability, and loss of autolysis. Proc Natl Acad Sci U S A. 1968 Jan;59(1):86-93. doi: 10.1073/pnas.59.1.86. PMID: 4385544; PMCID: PMC286006.
2. Lopez R, Garcia E, Garcia P, Ronda C, Tomasz A. Choline-containing bacteriophage receptors in Streptococcus pneumoniae. J Bacteriol. 1982 Sep;151(3):1581-90. doi: 10.1128/jb.151.3.1581-1590.1982. PMID: 7107560; PMCID: PMC220440.
3. Ware D, Watt J, Swiatlo E. Utilization of putrescine by Streptococcus pneumoniae during growth in choline-limited medium. J Microbiol. 2005 Oct;43(5):398-405. PMID: 16273030.

Line 355:
Typo 'mutations win the tacF gene…'

We have implemented the changes recommended by the reviewer.

Fig. 1:
It seems that the TacF structures in panels e and f/g are shown in different orientations. The cytosolic side is pointing up in panel e and down in panels f/g. This is confusing

We have changed the orientation of the figure for clarity.

Also, please add an arrow to panel f that shows the rotation direction and label TM helices 1 and 8 in the structure shown to the left.

We have implemented the changes recommended by the reviewer.

Sample assembly for cryo-EM:

The description of the BRIL–TacF:BAG2 complex lacks detail. Please include protein concentrations and incubation time prior to size-exclusion chromatography, which produced the cryo-EM sample.

We have implemented the changes recommended by the reviewer.

Line 413 – Protein expression:
Please include details on induction conditions (e.g., culture OD at induction).

We have implemented the changes recommended by the reviewer.

Figure 4:
The figure and its discussion would benefit from the following clarifications:
-additional details on the D39V and VL4012 strains

We thank the reviewer for the suggestion to clarify about details on the *S. pneumoniae* D39V and VL4012 strains. *S. pneumoniae* D39V is a laboratory-derived strain of the historically important serotype 2 clinical isolate D39, originally related to the strain used in studies by Oswald Avery and colleagues that demonstrated DNA as the genetic material. The D39V lineage represents a re-cultured, sequence-verified derivative that has been propagated through several laboratories before being established in the Veening lab (**see figure below**), as described in Slager et al., 2018 (Reference 119).

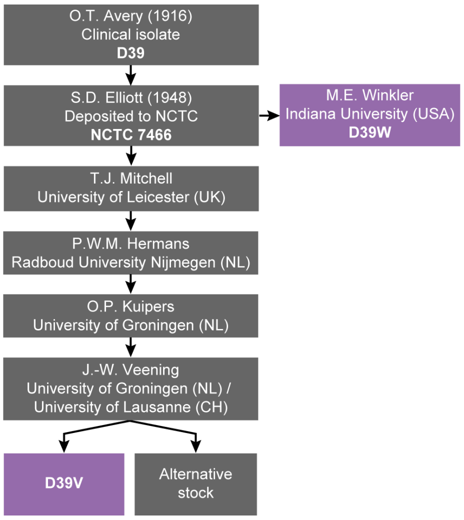

Figure. D39V strain history. *S. pneumoniae* serotype 2 strain D39 was isolated in 1916 by Oswald Avery and deposited in the National Collection of Type Cultures in 1948, then re-cultured multiple times before being established in the Veening lab as D39V.

VL4012 is a D39V derivative strain engineered with a dual inducible ectopic expression system for *tacF*. An ectopic wild-type *tacF* copy under an IPTG-inducible promoter (Plac) was inserted at the ZIP locus, and the native *tacF* locus was replaced with an antibiotic resistance marker to allow titration of wild-type expression. Mutant *tacF* alleles were inserted under an aTc-inducible promoter (Ptet) at the *bgaA* locus, enabling controlled expression of either wild-type or mutant *tacF.* We have added these details in the methods section. Line 657-658.

-larger, more legible labels
We have implemented the changes recommended by the reviewer.

-clarify whether R15 and R269 were tested, and if not, why.

The double mutant of residues R15 and R269 was not constructed due to limitations in the experimental setup and available resources. We have therefore modified the text to more clearly emphasize the hypothetical nature of the proposed model and to explicitly acknowledge that additional experiments will be required in future studies to further test and refine the proposed mechanism. See Line 367-370.

Overall, the figures are quite small, which makes it hard to read the labels without zooming in.

We have implemented the changes recommended by the reviewer.

Reviewer #4:

The current work by Cebrero et al. presents the structure and functional analysis of the Streptococcus pneumoniae TacF, a key flippase that monitors the phosphocholine content of teichoic acids (TA), using Cryo-EM, in vivo complementation assays, and in-silico molecular dynamics simulations. Due to the difficulty of obtaining a high-resolution reconstruction map for wild-type TacF, the cryo EM structure determination involved a clever way to first create fusion constructs of TacF with a marker “BRIL” and BRIL-binding synthetic antibody fragment BAG2. Of the six computationally (AF3) guided TacF constructs, BRIL9-TacF was pursued further for structure elucidation in nanodiscs, which resulted in a 3.63 Å resolution structure, with TacF structure in its inward-facing conformation in 2.5 Å resolution. The subsequent functional assays, evolutionary analysis, and MD simulations identified conserved motifs involved in substrate recognition and flippase activity. These investigations allowed the authors to propose a mechanistic model of the substrate recognition, binding, and flipping of PC-derived TA.
This work improves our understanding of the structure and mechanism of action of Streptococcus pneumoniae flippase TacF. While lipid II flippase MurJ structures have been extensively characterized, the lack of TacF structures poses a significant knowledge gap, and this work with the inward-facing TacF will be of great significance for the wider scientific community. Assessment of the work shows that the experimentations have been robust for both experiments and in silico investigations. However, a few points need to be addressed.

We appreciate the referee's thorough review of our manuscript and their thoughtful comments.

1) Substrate-binding Experiment: Was a TacF complexed with the substrate attempted for structure determination?

(See answer to reviewer #1). In summary, we attempted to obtain a high-resolution structure of a substrate-bound state. We invested significant effort in reconstituting the TacF-BRIL construct into nanodiscs containing lipid extracts of *S. pneumoniae* teichoic acids. Unfortunately, the reconstruction maps obtained yielded an average resolution of 8.5 Å, limiting analysis of the binding mode of teichoic acids to TacF (**See Figure below**). The volume obtained showed what we believe is a different conformational state, but due to the low resolution, it was not possible to reach a meaningful conclusion.

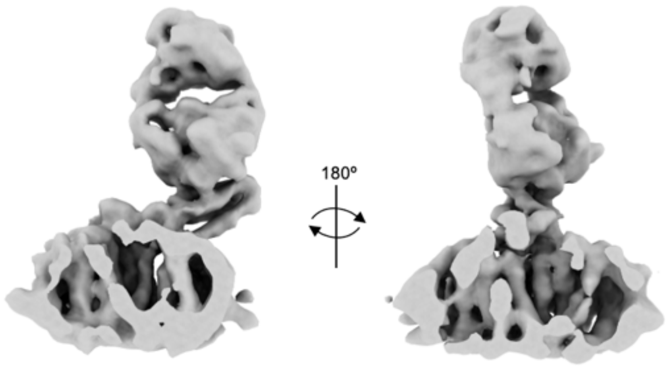

Figure. TacF reconstituted in nanodiscs inclusing teichoic acid lipid extracts. Sliced view of the 8.5Å reconstruction map of TacF:BRIL.

Computational: The authors describe the positioning of TA “near the lateral entrance”

between TM1 and TM8, but it's unclear whether the placement was done with molecular docking, or a randomized placement between TM1/TM8 with TA already pulled up, or a randomized placement firmly embedded in the lipid bilayer. The description of how the TA was initially placed is important to correctly interpret the TA-protein residue interactions in the context of the solved structure and the timescale of the simulations. The phosphate groups appear to be placed in the proximal cavity. Did the tail fit in the hydrophobic groove? While substrate-bound structures of TacF are likely to be different, the rationale for choosing the proximal site as the initial putative binding site is unclear, especially because of low sequence conservation at that site.

We thank the reviewer for raising this important point. The teichoic acid was placed taking into account two considerations: (i) TacF architecture: TacF and other MOP transporters exhibit a unique asymmetric structure, allowing only one side of the protein to fully open to the cytoplasm when in the inward-facing state (**See Figure below**). The side with the groove allows complete access from both the cytoplasmic and extracellular sides by opening and closing the entrance formed by TM1 and TM8 during alternating-access cycles (see black dashed lines). In contrast, the opposite side of the protein does not open toward the cytoplasm (minimally) but only toward the extracellular space, via the entrance formed by TM2 and TM7. On this side of the protein, the cytoplasmic entrance is obstructed by the bent TM7 (red square) in both inward- and outward-facing conformations. Therefore, since teichoic acid molecules would need to reach the central cavity when TacF is inward-facing, we decided to position it on the side that fully opens to the cytoplasm. (ii) Similarities with the lipid-II flippase, MurJ: MurJ has been extensively studied through mutagenesis and in-silico analyses, supporting that docking of lipid-II on the side of the groove is the most probable scenario[1,2]. Therefore, based on the similarities between TacF and MurJ in the groove and lateral entrance architecture, we believe that a similar binding of the undecaprenyl tail occurs in TacF.

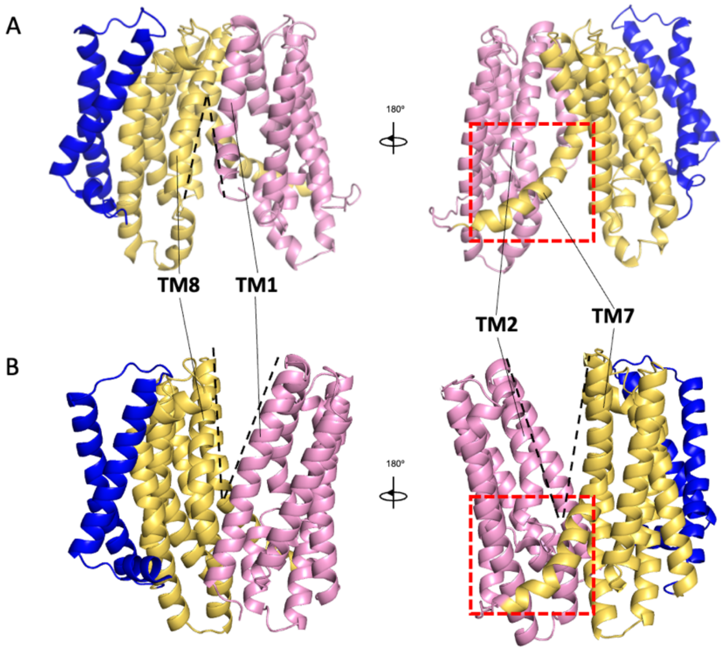

Figure. A. TacF (cryo-EM structure) in inward-facing state. **B.** TacF (AF3 Model) in outward-facing state.

1. Kuk AC, Mashalidis EH, Lee SY. Crystal structure of the MOP flippase MurJ in an inward-facing conformation. Nat Struct Mol Biol. 2017 Feb;24(2):171-176. doi: 10.1038/nsmb.3346. Epub 2016 Dec 26. PMID: 28024149; PMCID: PMC5382020.
2. Kuk ACY, Hao A, Lee SY. Structure and Mechanism of the Lipid Flippase MurJ. Annu Rev Biochem. 2022 Jun 21;91:705-729. doi: 10.1146/annurev-biochem-040320-105145. Epub 2022 Mar 23. PMID: 35320686; PMCID: PMC10108830.

Also, the time-dependent interaction map (Fig. S6) isn't very helpful without showing the binding poses. By 1 microsecond, the TA chain may have different clusters with varying residence times, and representative structures showing the interactions will be helpful. Since there was no movie or trajectory provided, it was difficult to see exactly what was done.

We have now provided a movie that shows 1000 ns of the simulation. The undecaprenyl chain indeed shows different conformations. Previous studies have shown that undecaprenyl lipids adopt multiple conformations in membranes, including bent V- and L-shaped states[1,2,3], consistent with the conformational ensemble observed in our MD simulations. We propose that the hydrophobic groove preferentially accommodates the undecaprenyl tail due to (i) its geometry, which is compatible with both V- and L-shaped conformations (**Fig. 3A**), and (ii) the extensive hydrophobic contacts formed with the polyisoprenoid chain due to its length. This extended interaction surface is expected to prolong the residence time of the undecaprenyl tail in the groove. See new text in Line 363-367. We have also added a new figure showing representative snapshots for the major clusters occupied by the undecaprenyl tail (**Suppl. Fig. 7A**). The representative snapshots of the clusters have be deposited in the Zenodo repository..

1. Mulholland S, Turpin ER, Bonev BB, Hirst JD. Docking and molecular dynamics simulations of the ternary complex nisin2:lipid II. Sci Rep. 2016 Feb 18;6:21185. doi: 10.1038/srep21185. PMID: 26888784; PMCID: PMC4758073.
2. Chugunov A, Pyrkova D, Nolde D, Polyansky A, Pentkovsky V, Efremov R. Lipid-II forms potential "landing terrain" for lantibiotics in simulated bacterial membrane. Sci Rep. 2013;3:1678. doi: 10.1038/srep01678. PMID: 23588060; PMCID: PMC3627190.
3. Kuk ACY, Hao A, Guan Z, Lee SY. Visualizing conformation transitions of the Lipid II flippase MurJ. Nat Commun. 2019 Apr 15;10(1):1736. doi: 10.1038/s41467-019-09658-0. PMID: 30988294; PMCID: PMC6465408.

2) Insights on substrate placement/binding as well as the interaction patterns in different but related systems may offer some guidance (e.g.,https://www.nature.com/articles/s41467-023-37702-7/figures/2; https://www.nature.com/articles/s41467-025-58196-5).

We thank the reviewer for this thoughtful suggestion. We have added new figures that show further insights into substrate placement/binding (**See Suppl. Fig 6C,D**).

3) Mutational studies of S. pneumoniae growth have been outlined in Figure 4. However, R15 and R269, which are suggested to play a key role in the initial recognition and interaction of TA, have not been considered. Wouldn't that provide strong insights into the presented mechanistic model?

Thanks to the reviewer for pointing this out. The double mutant of residues R15 and R269 was not constructed due to limitations in the experimental setup. Given our current situation, including the student who conducted the experiments graduating, we cannot conduct these experiments at this time. We have therefore modified the text to make the hypothetical

nature of the proposed model clearer and to explicitly acknowledge that additional experiments will be required in future studies to further test and refine the proposed mechanism. See Line 367-370.

4) Please provide the rationale (or a reference) for choosing the specific lipid composition in the methods. Also, describe whether it was an asymmetric bilayer (with corresponding compositions in the upper and lower leaflets).

We have included references to the lipid composition of *S. pneumoniae* in the results section, please see lines 131-133 and references 64 and 65. Regarding asymmetry, our nanodisc assembly protocols by detergent removal do not impose leaflet-specific lipid distributions. Consequently, lipid partitioning between leaflets arises from the assembly process rather than being an experimentally specified parameter. Importantly, our conclusions do not assume leaflet-specific lipid localizations.

5) Also mention the number of replicas and production lengths in the Methods.

We have now included detailed clarifications by adding a supplementary table detailing the specifics of all simulations performed in this work (See **Suppl. Table 3**).

Reviewer #5:

This manuscript provides a cryo-EM structure of S. pneumoniae TacF in lipid nanodiscs. TacF has been proposed to bind and translocate (flip) undecaprenol-phosphate-bound repeat units of teichoic acid across the plasma membrane. In addition to the structure, the authors perform sequence similarity analyses, site directed mutagenesis and molecular dynamic simulations to address the selectivity of TacF toward phosphocholine-substituted substrate that represents the mature building block of teichoic acid in several bacterial species. Aided by the detailed understanding of other flippases including MurJ and RFT1, a model is proposed in Figure 6 to describe substrate recognition and translocation across the membrane. It is interesting that the cryo-EM structure of TacF (without substrate) is that of the inward facing protein; such that the inward state is also the resting state prone to accepting the substrate. The authors also propose a common evolutionary origin between these proteins and distinct key features for phosphocholine containing substrates. Overall, the study is well conducted and informative.

We appreciate the referee's insightful feedback and constructive comments on our manuscript. In response, we have revised the paper, and we hope these changes address the concerns raised.

Comments:
1. Sup Fig 2: some sort of labeling should be used to delineate the BRIL segment from TacF.

We have made the corresponding changes to **Supp. Fig. 2**.

2. Figure 2: the authors analyze many sequences including some from Staphylococcus, Bacillus and Clostridia. This is a bit confusing. Most Staph/Bacillus strains share similar WTA and the ABC transporter complex TarGH is used to translocate fully polymerized WTA polymers. Are the authors comparing TacF to TarGH or rather to LtaA-like enzymes for the assembly of the LTA glycolipid anchor? Clostridia teichoic assembly pathways are not clearly delineated so it is difficult to understand from the cited references to what Clostridial proteins TacF is being compared. In general, references #23, 63-65, cited in this section do not particularly provide clarity. Perhaps, the authors are simply collecting all proteins of the MOP family retrievable from Gram positive genomes?

We thank the reviewer for the comment. Below we show the bacterial species that are included in Cluster III. The sequence similarity network (SSN) was generated using BLAST with an E-value cutoff of 5 and a maximum of 1,000 BLAST hits and limited to search in Gram-positive bacteria species. No length filtering was applied (minimum length 0; maximum length 50,000 residues). Network edges were filtered by E-value and represent pairwise relationships with at least 40% sequence identity between connected nodes. In total, the SSN shown in Figure 2 comprises 709 sequences. As the reviewer noted, it is notable that although the literature reports that most bacterial species in cluster III have type I teichoic acids, these species also appear to have genes encoding TacF-like proteins that share at least 30% identity with *Streptococcus pneumoniae* TacF. We speculate that these TacF-like proteins are flippases involved in uncharacterized biosynthetic pathways. The nature of these pathways and the function of these proteins will be investigated in the future.

We have clarified this in the manuscript. See Lines 195-199. Additional references that might clarify these points are also included.

**Bacterial species in Cluster III**
Bacillus anthracis.
Bacillus cereus.
Bacillus fungorum.
Bacillus manliponensis.
Bacillus mycoides (strain KBAB4)
Bacillus pacificus.
Bacillus paranthracis.
Bacillus pseudomycoides.
Bacillus thuringiensis serovar pingluonsis.
Bacillus timonensis.
Bacillus toyonensis.
Bacillus tropicus.Bacillus sp. L27.
Bacillus wiedmannii.
Candidatus Faecivivens stercoripullorum.
Candidatus Merdivicinus excrementipullorum.
Clostridium algidicarnis DSM 15099.
Clostridium amylolyticum.
Clostridium beijerinckii
Clostridium botulinum.
Clostridium butyricum.
Clostridium chromiireducens.
Clostridium diolis.
Clostridium disporicum.
Clostridium folliculivorans.
Clostridium frigidicarnis.
Clostridium fungisolvens.
Clostridium manihotivorum.
Clostridium neonatale.
Clostridium perfringens.
Clostridium polyendosporum.
Clostridium puniceum.
Clostridium putrefaciens.
Clostridium saccharobutylicum.
Clostridium saccharoperbutylacetonicum N1-4
Clostridium taeniosporum.
Clostridium uliginosum.
Fumia xinanensis.
Inconstantimicrobium porci.
Mesobacillus boroniphilus.
Mesobacillus selenatarsenatis.
Neobacillus massiliamazoniensis.
Neobacillus novalis.
Neobacillus thermocopriae.
Niallia endozanthoxylica.
Olsenella profusa F0195.
Paenibacillus graminis.

3. Line 249: add ‘Figure 4C’ at the end of the sentence.

Thank you. We have made this change.

4. Figure 4b: in this panel, it would be nice to display R152, F227, Y172, T25, Y38 and some of the other substitutions tested in panels c,d,e.

We have added a new supplementary figure that includes all these residues, **Suppl. Fig. 8A**. We also included a note about this figure in the **Figure 4** legend.

5. Figure 4/Suppl Figure 7: Residues selected for mutations should all be accounted for in the text describing these data. For example, the authors should explain how combinations of residues to generate double and triple mutations were selected.

Thanks to the reviewer for pointing this out. Given the large number of residues in the cavity that could contribute to teichoic acid binding, we selected combinations of mutations to maximize coverage of key residues while minimizing the number of constructs required. We acknowledge the reviewer's point and agree that, in principle, many additional combinations could be generated. However, the construction and characterization of these mutants are highly challenging and technically demanding, which limits the combinations we can present in this study.

As another example, the authors do not show the effect of F227A alone only in combination with other substitutions. Does F227A behave like wild type?

Thanks to the reviewer for pointing this out. We have added the results for the F227A mutant in **Suppl. Fig 8B,C**.

6. Figure 5: here the authors compare 138,448 sequences. How do these sequences relate to those in Figure 2?

We thank the reviewer for raising this point. The sequence datasets used to generate the sequence similarity network (SSN) in Figure 2 and to perform the direct coupling analysis (DCA) in **Figure 5** were generated using different search algorithms and different search databases and therefore do not have accession ID overlap. The dataset used to construct the SSN in **Figure 2** was generated using BLAST against the UniProt database, returning 1,000 sequence hits, of which 709 were plotted in the SSN. In contrast, jackHMMER was used in the DCA to search the UniRef90 database, which clusters UniProt sequences at 90% identity and uses cluster representatives. From this search, 138,448 sequences were obtained and used for the DCA, which inherently requires a more diverse dataset to detect coevolutionary patterns. As such, the SSN and DCA datasets were curated to serve the purpose of their respective analyses and thus do not share any accession IDs.
An all vs. all pairwise sequence comparison of the two datasets using MMseqs2 showed that each SSN sequence had a detectable match within the DCA sequence dataset with a mean sequence identity of 28.0% and a standard deviation of 11.9%. This indicates that both datasets sample the same homologous family but that the DCA dataset includes more divergent homologs.

7. Figure 6: do the authors propose that the enzyme returns to the inward facing state spontaneously? Is the undecaprenol-intermediate simply pushed outside of the channel? Could the action of TarP/TarQ facilitate the return to the inward state? At this stage, speculations are still allowed.

We agree with the reviewer that there is room for speculation about how TacF transitions between conformational states and the potential role that TarP/TarQ could play in this

process. We modified the text to include a hypothesis about how this occurs. See Line 388-390.

Reviewer #4 (Remarks to the Author):

The revised manuscript by Cebrero et al. addressed the concerns pointed out in the initial round of review. Here are some minor comments about the modeling of the outward-facing conformation of TacF bound to teichoic acid (TA).

We appreciate the referee's insightful feedback and constructive comments on our manuscript.

The authors used AF2 to model the outward-facing conformation of TacF and performed molecular docking of TA. The fragment docking approach taken by the authors seems to be appropriate, but the following are the comments/suggestions on the reconstruction of the molecule post-docking:

1. Since Glide SP only allows the ligand to be fully flexible and not the protein, manual placement of the TA tail to its polar headgroup post docking may need a short minimization to fully allow the TA molecule to be accommodated in the protein and account for side chain movements within the binding site that would otherwise not be accounted for in SP mode. Perhaps performing docking of the TA tail in the vicinity of the polar head group using the induced fit mode would avoid piecewise reconstruction.

We thank the reviewer for bringing up this point. We agree with the reviewer that accounting for protein flexibility is crucial. In our workflow, we performed energy minimization of the TacF-TA complex immediately after manually placing the TA tail. This was followed by 3 replicates of 300 ns all-atom Molecular Dynamics (MD) simulations (see methods section, lines 633-635). MD-based relaxation provides a comprehensive sampling of the conformational landscape and side-chain adjustments, in contrast to Induced Fit Docking (IFD), which misses much of the system's flexibility.

We thank the reviewer for suggesting docking the TA tail. We have done this using IFD. Given the high flexibility and conformational freedom of the TA tail, the docking did not produce well-converged or physically intuitive poses.

2. An alternative, or an additional exercise, would be cofolding of TA and TacF using cofolding methods like Boltz-2 and Chai-2/Openfold3 that would enable AI-based prediction of not just the conformations but also the affinity of TA bound to TacF. Have the authors explored this?

We thank the reviewer for this insightful suggestion. We have explored this approach and performed co-folding predictions using Boltz-2, Chai-1, and OpenFold3. The predicted binding poses from these AI-based methods showed: (i) The general binding pose of the TA is highly consistent with the reported results, as the undecaprenyl tail remains interacting with the hydrophobic groove, and the TA headgroup stays inside the central cavity (**See Figure below**); (ii) The TA headgroup maintain interactions with the key residues in the central cavity, as described by our initial outward-facing (OF) conformation model and MD simulations results. (**See Figure below**)

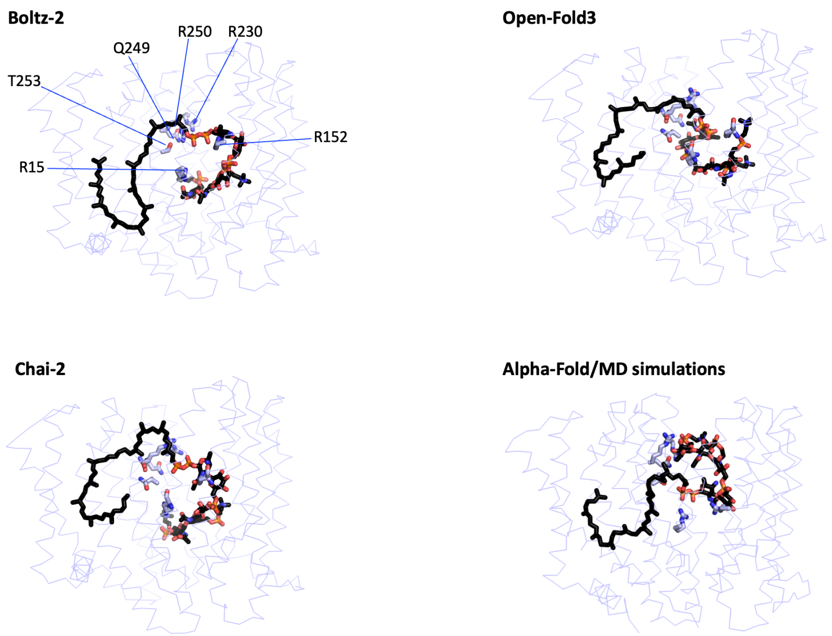

Figure: Comparison of different predicted models of TacF outward-facing state bound to a teichoic acid (TA) molecule. TacF, blue ribbons. TA, black sticks. Central cavity residues are shown as blue sticks.
